# The intracellular lipid-binding domain of human Na^+^/H^+^ exchanger 1 forms a lipid-protein co-structure essential for activity

**DOI:** 10.1038/s42003-020-01455-6

**Published:** 2020-12-03

**Authors:** Ruth Hendus-Altenburger, Jens Vogensen, Emilie Skotte Pedersen, Alessandra Luchini, Raul Araya-Secchi, Anne H. Bendsoe, Nanditha Shyam Prasad, Andreas Prestel, Marité Cardenas, Elena Pedraz-Cuesta, Lise Arleth, Stine F. Pedersen, Birthe B. Kragelund

**Affiliations:** 1 Structural Biology and NMR Laboratory, Department of Biology, University of Copenhagen, Ole Maaløes Vej 5, DK-2200 Copenhagen N, Denmark; 2 Cell Biology and Physiology, Department of Biology, University of Copenhagen, Universitetsparken 13, DK-2100 Copenhagen Ø, Denmark; 3 Niels Bohr Institute, University of Copenhagen, Universitetsparken 5, 2100 Copenhagen Ø, Denmark; 4 Biofilms Research Center for Biointerfaces, Malmö University, Per Albin Hanssons Väg 35, 214 32 Malmö, Sweden

**Keywords:** Biophysics, Cell biology, Computational biology and bioinformatics, Structural biology

## Abstract

Dynamic interactions of proteins with lipid membranes are essential regulatory events in biology, but remain rudimentarily understood and particularly overlooked in membrane proteins. The ubiquitously expressed membrane protein Na^+^/H^+^-exchanger 1 (NHE1) regulates intracellular pH (pH_i_) with dysregulation linked to e.g. cancer and cardiovascular diseases. NHE1 has a long, regulatory cytosolic domain carrying a membrane-proximal region described as a lipid-interacting domain (LID), yet, the LID structure and underlying molecular mechanisms are unknown. Here we decompose these, combining structural and biophysical methods, molecular dynamics simulations, cellular biotinylation- and immunofluorescence analysis and exchanger activity assays. We find that the NHE1-LID is intrinsically disordered and, in presence of membrane mimetics, forms a helical αα-hairpin co-structure with the membrane, anchoring the regulatory domain vis-a-vis the transport domain. This co-structure is fundamental for NHE1 activity, as its disintegration reduced steady-state pH_i_ and the rate of pH_i_ recovery after acid loading. We propose that regulatory lipid-protein co-structures may play equally important roles in other membrane proteins.

## Abstract (web summary)

Hendus-Altenburger et al. provide biochemical, structural and functional information on the lipid interaction domain (LID) of the Na+/H+ Exchanger 1 (NHE1). They find that NHE1-LID is intrinsically disordered, but, when allowed to interact with a lipid membrane, forms a helical αα-hairpin, stabilized by hydrophobic and electrostatic interactions. This co-structure is fundamental for NHE1 activity, giving insight into membrane protein regulation via disordered domains.

## Introduction

Mechanistic understanding of membrane proteins has increased tremendously in recent years due to structural insights facilitated by the improvements of cryoEM resolution. In addition to the highly structured regions amenable to such analyses, intrinsically disordered N- and C-terminals appear frequently in the human transmembrane proteome^1^. Such intrinsically disordered regions (IDR) play key roles in membrane protein function^2^, thus adding an additional layer of complexity to the mechanistic understanding of these proteins. IDR in membrane proteins can be hundreds of residues long^1^, but they are absent or silent in most structure studies and generally neglected in the understanding of function. While disordered regions in isolation can be studied and understood at the atomic level^3^, recent studies have shown that IDRs in membrane proteins engage in interactions with the membrane^4–7^, often of dynamic nature, constituting a huge methodological challenge. Furthermore, changes in membrane composition are emerging as physiological and pathophysiological relevant mechanisms modulating membrane protein function^8–10^. Collectively, this shows the necessity of uncovering how disordered regions in membrane proteins cross-talk with and engage in lipid:protein co-structures relevant to function.

The Na^+^/H^+^-exchanger isoform 1 (NHE1, SoLute Carrier 9A1 (SLC9A1)) is a membrane protein with long IDRs, and is a major regulator of intracellular pH (pH_i_) in essentially all mammalian cells studied. NHE1 is activated by intracellular acidification, as well as by cytokines, growth factors, osmotic cell shrinkage, and cell-matrix adhesion^11,12^. NHE1 dysregulation has been linked to several pathological conditions, with particularly important roles in cardiovascular diseases and cancer^12–14^. The transmembrane domain of NHE1 is mandatory for ion transport, whereas its ∼300-residue long, regulatory C-terminal cytosolic tail (*ct*) controls the pH_i_ set point of the transporter and is required for allosteric NHE1 regulation^11,12,15^. The tail serves as an interaction hub for many binding partners including constitutively bound calcineurin homologous proteins (CHPs)^16–18^ and harbors several predicted and confirmed regulatory phosphorylation sites^11^. Deletion of most or all of the NHE1*ct* strongly reduces ion transport activity, and shifts activation of NHE1 by protons to more acidic values^15,19^.

The NHE1*ct* can be divided into four structural subdomains (subdomains A-D)^11^ largely corresponding to four previously described functional domains^20^. Subdomain A and C are predicted to be helical and to recruit most of the confirmed interaction partners, whereas B and D, located between the two folded domains and at the distal tail, respectively, have high scores for intrinsic structural disorder, properties that were confirmed experimentally for the distal 130 residues^21,22^. The proximal third of subdomain A corresponding to R516-G539 in the human (h)NHE1, forms an α-helix in complex with CHP1, −2 or −3^16,17,23^. However, molecular details of the structure, dynamics and interactions of the remaining two-thirds of subdomain A are lacking, hampering understanding of the function of this domain.

The activity of NHE1 is dependent on different types of lipophilic compounds, including ATP^24,25^ and various phosphoinositides^26,27^. In the hNHE1*ct*, two phosphatidylinositol-4,5-bisphosphate (PI(4,5)P_2_)-binding sites have been identified in subdomain A: KKKQETKR_509-516_ (site I) and RFNKKYVKK_552–560_ (site II), flanking the CHP binding site^27^. The abundance of [KR]-residues and the hydrophobic character of the surrounding residues bears resemblance to other [KR]-motifs involved in phosphoinositide binding^2,28^. The functional importance of NHE1:PI(4,5)P_2_ interaction was underscored by the finding that in kidney glomerular injury, accumulating amphipathic long-chain acyl-CoA (LC-CoA) metabolites competed with PI(4,5)P_2_ for NHE1 binding, leading to reduced NHE1 function and consequent increased susceptibility of proximal tubule cells to apoptosis^8^.

The reported lipid-binding portfolio of the hNHE1*ct* includes several negatively charged membrane lipids, ranked here by their apparent affinities: phosphatidylinositol (3,4,5)-trisphosphate (PI(3,4,5)P_3_) > phosphatidylinositol-bisphosphates (PI(3,4)P_2_, PI(4,5)P_2_) > and -monophosphates (PI) ≈ phosphatidic acid (PA) > phosphatidyl serine (PS)^26^. The second PI(4,5)P_2_-binding site plus an additional 46 residues, G542–P598, interacts with phorbol esters /diacylglycerol (PEs/DAG) in a transport regulatory manner enhancing membrane interaction^29^, and binds ATP in competition with PI(4,5)P_2_^30,31^. This region was accordingly defined as the lipid interaction domain of NHE1, i.e. the NHE1-LID. The NHE1-LID-phospholipid interaction was shown to be pH dependent in a manner relying on a cluster of histidine residues between the PI(4,5)P_2_ binding sites^32^, and membrane interaction was suggested to be mainly electrostatically driven^26^. The C-terminal tail of the related isoform SLC9A3 (NHE3) also interacts with membrane lipids^33,34^, supporting a general role for membrane interaction in the SLC9A family. However, the molecular details of the membrane interaction, including its structure and potential conformational changes in response to changes in membrane composition and other local microenvironmental changes, as well as the driving forces and sequence determinants for the interaction, remain essentially unstudied.

Here, we decompose the structure of the hNHE1-LID constituting residues G539-G593 (hereafter denoted NHE1-LID_539-593_). We delineate two structurally distinct but integrated sub-regions of the NHE1-LID, which, in absence of negatively charged membrane mimetics, are intrinsically disordered. In the presence of negatively charged lipids, NHE1-LID forms a folded, helical co-structure with the membrane, organizing itself in a dynamic helix-hairpin-helix (αα-hairpin) conformation with the hydrophobic, most C-terminal part penetrating into the headgroup region of the lipid bilayer. Disintegration of the NHE1-LID structure strongly inhibits NHE1-mediated recovery of pH_i_ after an acid load. This structure and its sensitivity to membrane lipid composition and to physico-chemical factors such as pH make the NHE1-LID central to understanding NHE1 regulation. We propose that such membrane:protein co-structures are likely to be important for many other membrane proteins with IDRs and relevant to their regulation.

## Results

### The NHE1-LID is intrinsically disordered with two transiently populated α-helices

**Fig. 1:**
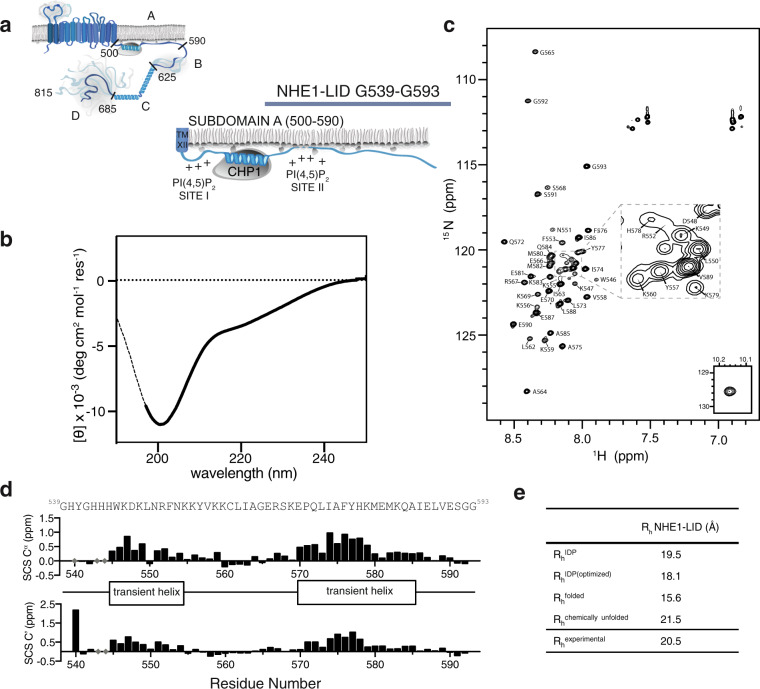
The NHE1-LID is intrinsically disordered. **a** Schematic architecture of human NHE1 indicating the subdomains A-D in the tail and with a zoom on the lipid interaction domain (LID) within subdomain A. **b** Far-UV CD spectrum of the NHE1-LID_539-593_ in H_2_O, pH 6.0. **c** ^15^N,^1^H-HSQC spectrum of NHE1-LID_539-593_ in the absence of membrane mimetics (pH 6.4). **d** Secondary chemical shifts (SCS) of C^α^ and C’ from backbone assignments of NHE1-LID showing two transient and lowly populated helices. **e** Radius of hydration, *R*_*h*_ of NHE1-LID_539-593_ from different scaling laws^37^ and experimentally determined using diffusion NMR (*R*_*h*_^*experimental*^).

Despite many investigations underscoring the relevance of various lipids for NHE1 function, and the previous identification of the NHE1-LID as a key region for NHE1 regulation, no structural data exist for this part of NHE1 (Fig. 1). To enable atomic resolution insight, we employed an ensemble of biophysical methods to delineate the structural properties of the 55-residues long NHE1-LID from hNHE1, encompassing residues G539-G593 (NHE1-LID_539-593_). The borders of the NHE1-LID_539-593_ were chosen from a structural perspective, starting just after the CHP1-binding helix (N-terminally), and ending before the disordered subdomain B (C-terminally), Fig. 1a.

Produced in isolation, NHE1-LID_539-593_ was highly prone to aggregation in phosphate buffer, in the presence of salt, or at a pH above 6.5. It only stayed in solution in 20 mM borate buffer, pH ≤6.4 or in pure water. Under these conditions, far-UV circular dichroism (CD) spectroscopy analysis of NHE1-LID_539-593_ revealed a dominantly disordered chain with very low content of helical structures, as evident from the negative molar ellipticity at 200 nm and 222 nm (Fig. 1b). Supporting this, the ^1^H-^15^N-HSQC nuclear magnetic resonance (NMR) spectrum of NHE1-LID_539-593_ showed low dispersion of signals in the proton dimension (Fig. 1c), another characteristic of a disordered protein.

To identify the location of transient secondary structures, which can be extracted from an NMR chemical shift analysis^35,36^, the NMR resonances of the NHE1-LID_539-593_ backbone atoms were assigned (86% coverage) and secondary chemical shifts (SCSs) calculated for C^α^ and C’ nuclei using peptide-based random coil shifts^35,36^. From the consecutive positive SCSs (Fig. 1d), two regions of transient α-helical structure, each populated by 20–30%, were identified: H540-F554 and E570-I586 (Fig. 1d). Finally, the hydrodynamic radius, *R*_h_ of NHE1-LID_539-593_ was determined from NMR diffusion measurements. Compared to theoretical values calculated for a chain of different properties^37^, NHE1-LID_539-593_ had an expanded dimension expected for an IDR (Fig. 1e).

Taken together, these data show that the LID region of NHE1 is disordered in the absence of lipids and that its solubility is highly sensitive to changes in ionic strength and pH. The disordered region populates two transient α-helices in an overall largely extended chain.

### Subdomain A of NHE1ct interacts with a broad range of lipids

**Fig. 2:**
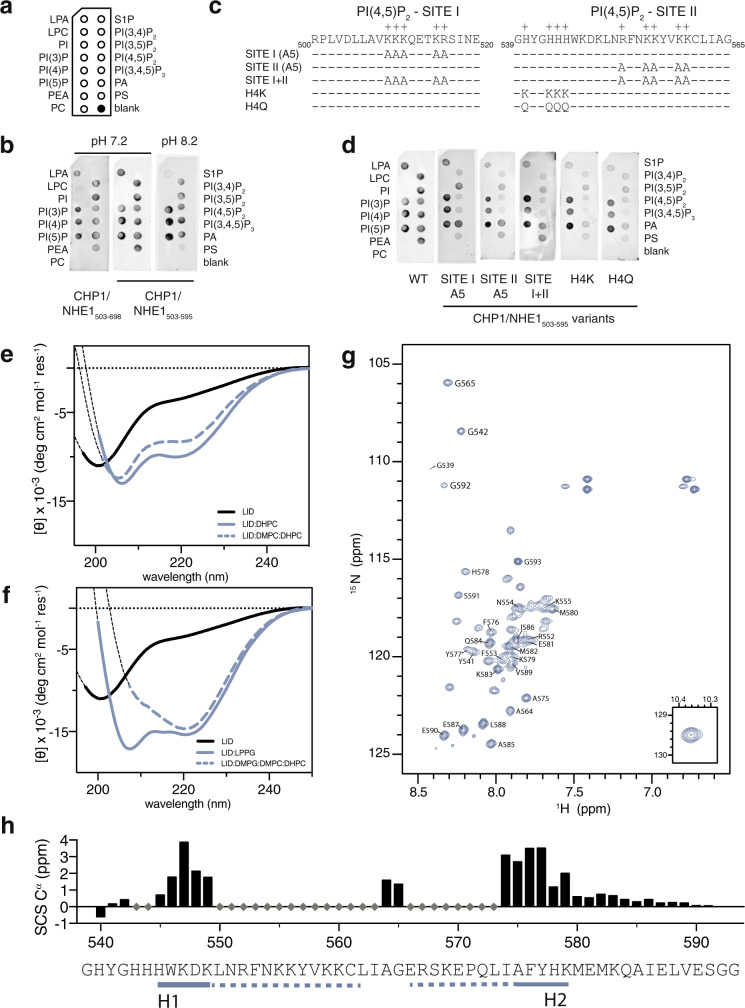
The NHE1-LID binds different lipids with induction of helical structures. **a** Position of individual lipids on the dot blot membrane. **b** Lipid binding profile of the CHP1/NHE1_503-595_ and CHP1/NHE1_503-698_ at pH 7.4 and 8.4. **c** Variants of NHE1 used to test lipid binding specificity. **d** Effect of various mutations on CHP1/NHE1_503-595_ lipid binding. **e** Far-UV CD spectra of NHE1-LID_539-593_ in DHPC detergent (color) and in DMPC:DHPC bicelles (dashed color). The CD spectrum of NHE1-LID_539-593_ in water is shown in black. **f** Far-UV CD spectra of the NHE1-LID in anionic bicelles consisting of DMPG:DMPC:DHPC (dashed color) and in 2% LPPG (color). The CD spectrum of NHE1-LID_539-593_ in water is shown in black. **g** ^15^N,^1^H-HSQC spectrum of NHE1-LID_539-593_ in 2% LPPG, 320.15 K. **h** SCSs of C^α^ of the NHE1-LID in 2% LPPG with two highly populated helices, H1 and H2, indicated below the sequence.

To determine if the NHE1-LID_539-593_ constitutes the major lipid interaction region of NHE1*ct* and to address its lipid specificity, we recombinantly produced two NHE1*ct* variants; NHE1_503–595_ (subdomain A) and NHE1_503–698_ (subdomain A-C). These proteins were produced in complex with the obligatory NHE1 binding partner CHP1 as this increased solubility. In lipid overlay assays, CHP1/NHE1_503–595/698_ bound to essentially all negatively charged lipids tested, including mono-, bis-, and tri-phosphoinositides as well as to PS, PA, and lyso-PA (LPA), Fig. 2a, b. There were no apparent qualitative differences in lipid binding between the two NHE1*ct* length variants, and although subdomain B and C may also be able to bind the same lipids, the complete lipid binding profile is fully represented by subdomain A. Increasing pH from 7.2 to 8.2 abolished binding to PS and LPA, but retained binding to all other tested lipids, suggesting a low lipid specificity of the NHE1-LID and a pH sensitivity towards only certain lipids (Fig. 2b). Notably, given their pKa values outside the tested pH range, the protonation state of PS and LPA does not change, indicating that the observed change in binding is to be found on the protein level. To delineate if the two known PI(4,5)P_2_-binding regions^27^ and the histidine rich stretch (HYGHHH_540–545_)^32^ were required for lipid binding, we prepared five variants of NHE1_503–595_ in which each site was mutated individually, using alanine substitution of the basic residues of the two PI(4,5)P_2_-binding sites and glutamine or lysine substitutions of the histidines (Fig. 2c). Neither mutations of the basic clusters individually or in combination, nor mutations of the histidines, abolished lipid binding. Yet, the mutations reduced the apparent binding to phosphatidylinositol bis- and tri-phosphates, as well as to PS, whereas binding to phosphatidylinositol mono-phosphates was almost unaffected (Fig. 2d). Thus, mutation of the positive charges did not abolish binding, but changed lipid preference. Furthermore, changing the charged state of the histidine cluster by mutations to either lysine (H4K) or glutamine (H4Q) had the same abolishing effect on the binding to LPA, PS and the phosphatidylinositol bis- and tri-phosphates, pointing towards a specific interaction (Fig. 2d). As many differently charged lipid species are recognized by NHE1-LID, with variable sensitivity to changes in pH, charge, and mutations, this also suggests that electrostatics alone cannot fully account for the interaction profile.

These results confirm that the main lipid binding ability of NHE1*ct* resides in subdomain A, i.e., residues 503–595. The broad lipid specificity demonstrated here supports earlier findings but further suggests that binding only partially depends on electrostatics. Moreover, changes in pH as well as the protonation state of the NHE1-LID modulated lipid species preference.

### The NHE1-LID_539-593_ interacts with anionic membranes and forms helical structures

We next used CD spectroscopy to investigate if and how various detergents, lipids and membrane mimetics would affect the secondary structure of NHE1-LID_539-593_. To allow us to assess lipid/detergent sensitivity as well as impact on variations in structural propensity, a variety of membrane mimetics were employed. Addition of zwitterionic 1,2-dihexanoyl-sn-glycero-3-phosphocholine (DHPC) detergent micelles to NHE1-LID induced the formation of distinct helical structure, populated on average ∼20% as judged from the increased negative ellipticity at 208 nm and 222 nm (Fig. 2e). A similar, albeit less populated helical structure formed when zwitterionic bicelles composed of DHPC: 1,2-dimyristoyl-sn-glycero-3-phosphocholine (DMPC) mixtures were used (Fig. 2e). Introducing negatively charged lipids in these bicelles in the form of 1,2-dimyristoyl-*sn*-glycero-3-phosphorylglycerol (DMPG) (DMPC:DMPG at 70:30 mol%) further changed the structure of the LID_539-593_ (Fig. 2f). Compared to the CD profile in Fig. 2e, DMPG increased the helical population to ∼40% and caused the θ_222nm_/θ_208nm_ ratio to become > 1. The latter is indicative of formation of coiled-coil structure^38^ compatible with a helix-hairpin-helix (αα-hairpin) structure, but could also reflect signals stemming from two aromatic sides in a particular orientation (T-stack)^39^, or a combination of the two.

As bicelles are not readily compatible with optimal NMR analyses, we next sought to identify a suitable detergent that would induce the formation of similar helical content in NHE1-LID. In 2% (w/v) 1-palmitoyl-2-hydroxy-*sn*-glycero-3-phosphoglycerol) (LPPG), a detergent used successfully for NMR analyses of membrane proteins^40^, the helicity of NHE1-LID was comparable to that in anionic bicelles, albeit the θ_222nm_/θ_208nm_ ratio was below one and less compatible with the coiled-coil structure (Fig. 2f). To optimize the resolution in the NMR spectra we adjusted the temperature to 320 K. The NMR signals of the NHE1-LID in the presence of LPPG were clearly upfield shifted (lower ppm) compared to the absence of LPPG (Supplementary Fig. S1) and were broader indicating dynamic helical structures (Fig. 2g). Although many of the signals in the triple resonance spectra were very weak as a result of dynamics, we achieved assignment of 49% of the backbone atoms in 2% LPPG (Fig. 2h) with SCSs of the C^α^ and C’ indicating strongly stabilized helical structures in the membrane bound state of NHE1-LID (SCS > 3 ppm). Positive SCSs indicated a helical region spanning H545-K579, but because of the lack of signals in the triple resonance spectra, we were unable to determine the remaining helix borders. Residues AG_563–564_ had a lower SCS of C^α^ of 0.8 ppm, suggesting that they populate a turn structure^41^. A smaller population (< 5%) of lower helix propensity was observed for residues in the very C-terminal end of the NHE1-LID (A584-E590).

Collectively, these results indicate that membrane association induces a helical folded state of NHE1-LID_539-593_ and that the helix population is increased by anionic lipids. More realistic membrane model systems as bicelles further stabilized the helicity, suggesting the formation of an αα-hairpin structure that remained dynamic on the membrane.

### The NHE1-LID_539-593_ is an interdependent entity with bipartite behavior

**Fig. 3:**
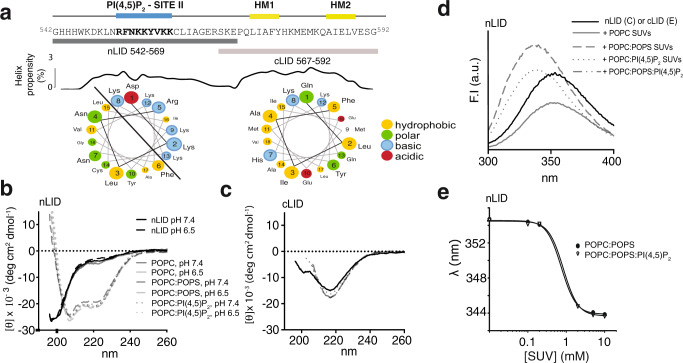
A bipartite structure of the NHE1-LID. **a** Sequence of the NHE1-LID with indicated peptide regions corresponding to nLID_542-569_ and cLID_567-592_ and with Agadir prediction of helicity and helical wheel representations^94^. The basic PI(4,5)P_2_ Site II (blue) and hydrophobic motifs (HM1, HM2) (yellow) indicated above. **b** Far-UV CD spectra of nLID_542-569_ alone and in the presence of various lipids and at two different pH values. **c** Far-UV CD spectra of cLID_567-592_ alone and in the presence of various lipids. **d** Fluorescence emission spectra of nLID_542-569_ alone and upon addition of POPC/POPS and POPC/POPS/PI(4,5)P_2_ SUVs. **e** Center of spectral mass analysis of nLID_542-569_ fluorescence emission spectra from a SUV titration series revealed an apparent membrane affinity of 0.8 mM for nLID_542-569_.

We next explored the individual contributions of the identified N- and C-terminal helical regions to the properties of the NHE1-LID_539–593_. Two overlapping peptides were designed and denoted nLID (G542-K569) and cLID (R567-G592) (Fig. 3a). Helical wheel representations predicted a strong amphipathic character of nLID_542–569_ with basic residues localizing to one side, in marked contrast to the overall hydrophobic cLID_567–592_. The nLID contains the basic residues involved in the site II PI(4,5)P_2_ binding site, while the cLID contains two hydrophobic motifs LIAFY_573–577_ (HM1) and AIELV_585–589_ (HM2) (grand average for hydropathy (GRAVY) score for the entire NHE1-LID_542–592_: −0.89; for LIAFY_573–577_: 2.32 and for AIELV_585–589_: 2.16^42^ (Fig. 3a)).

In the absence of lipids, nLID_542–569_ was fully water soluble and highly disordered with a minimum CD ellipticity at 190 nm and no shoulder at 222 nm (Fig. 3b). In marked contrast, cLID_567–592_ was only soluble at very low concentrations and displayed a mixed CD profile of coil and β-strand conformation with a broad minimum at 216 nm (Fig. 3c). This points towards an aggregated state of the isolated cLID_567–592_, fully consistent with its overall hydrophobic nature. This suggests that the NHE1-LID in its entirety has an internal chaperone function such that the more soluble nLID solubilizes the less soluble cLID, preventing formation of non-native, aggregated structures.

We next addressed how nLID_542–569_ and cLID_567–592_ would respond to the presence of membrane mimetics. The most abundant cellular lipid, 1-palmitoyl-2-oleoyl-glycero-3-phosphocholine (POPC), was used as template to study the effect of less abundant negatively charged lipids. Among the different lipids seen to interact with NHE1-LID in the lipid overlay assays, we chose two negatively charged and more abundant lipids in the inner leaflet of mammalian plasma membranes, 1-palmitoyl-2-oleoyl-glycero-3-phosphoserine (POPS) and phosphatidylinositol-4,5-bisphosphate PI(4,5)P_2_. No change in the CD profile of nLID_542–569_ was observed upon addition of small unilamellar vesicles (SUVs) made solely of POPC, as expected. Instead, the presence of 20 mol% POPS, or/and 1 mol% PI(4,5)P_2_, induced a dominant α-helical CD profile (Fig. 3b). No distinct additive effect was observed for SUVs containing both PI(4,5)P_2_ and POPS. In the same samples, the intrinsic fluorescence of W546 of nLID increased in intensity and blue-shifted from 354 nm to 339 nm upon addition of SUVs containing either POPS or PI(4,5)P_2_, indicating transition of W546 into a less polar environment (Fig. 3d). The apparent affinity of nLID for POPC/POPS SUVs was extracted from a titration series with an nLID concentration of 30 μM and increasing lipid concentration (Fig. 3d). We noted strong clouding within the transition phase (from 0.5 to 5 mM lipids) that resolved by further lipid addition, indicating temporary aggregation. A global fit of the fluorescence data gave an apparent affinity (*K*_*d*_^*app*^) of 0.8 ± 0.1 mM of nLID for POPC/POPS (Fig. 3e). However, the affinity of the entire LID in cellular context is most likely higher, given the additional hydrophobic residues of the cLID, the anchoring of the LID to the membrane by the TM domain, and the natural composition and curvature of the plasma membrane.

As the pH range of NHE1 activation (<pH 7.0)^12^ is similar to the range in which PI(4,5)P_2_ is known to titrate^43,44^ and the nLID contains four histidines, the NHE1-LID could potentially serve as a pH sensor via membrane constituents, as indeed previously shown for the histidine cluster (HYGHHH_540–545_) in a cellular context^32^. However, the CD spectra of nLID in the presence of SUVs of various lipid-content were largely independent of changes in pH from 4.7 to 8.4 with a slight decrease (∼10%) in helicity below pH 6 (Fig. 3b, Supplementary Fig. S2). This indicates that the nLID_542–569_ stays dominantly helical independent of the pH changes within the physiological range that would normally activate NHE1^12^.

When investigating the cLID_567–592_ in the presence of various SUVs (Fig. 3c) we observed no changes in average secondary structure. In all cases the structural profile remained reminiscent of an aggregated β-structure, with a minimum at 218 nm which became even more pronounced upon addition of negatively charged lipids.

These results highlight the markedly different properties of the two parts of NHE1-LID in vitro and show that the solubility, structure and membrane interaction of the hydrophobic C-terminal LID, cLID_567–592_, are strongly dependent on the presence of the amphipathic N-terminal region of the NHE1-LID_539-593_. Furthermore, membrane interaction of the nLID_542–569_ is mediated by negatively charged lipids and leads to the formation of a pronounced helical structure, which is not detectably altered by changes in pH.

### Hydrophobic residues drive membrane-induced structures of the C-terminal part of the NHE1-LID

**Fig. 4:**
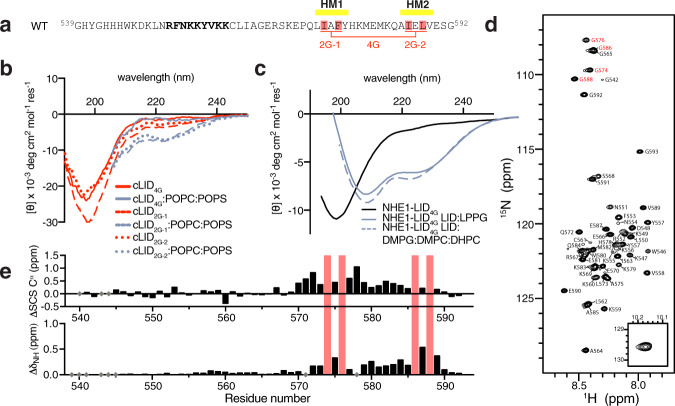
Hydrophobicity in the H2 region drives membrane interaction of the NHE1-LID. **a** Three different variants of the NHE1-LID_539-593_ were analyzed, NHE1-LID_539-593-2G-1_ (I574G, F576G in HM1), NHE1-LID_539-593-2G-2_ (I586G, L588G in HM2) and NHE1-LID_539-593-4G_ (I574G, F576G, I586G, L588G), as indicated. **b** Far-UV CD spectra of cLID_567-592_ peptides with glycine mutations in various lipids. **c** Far UV CD spectra of NHE1-LID LID_539-593-4G_ alone (black) and in the presence of 2% LPPG (color) and DMPG:DMPC:DHPC bicelles (dashed color). **d** ^15^N,^1^H-HSQC spectrum of NHE1-LID LID_539-593-4G_ in H_2_O, pH 6.5. **e** Differences in SCS between NHE1-LID_539-593_ and NHE1-LID_539-593-4G_ in the absence of membrane mimetics. Top: ΔSCSs of C^α^, bottom: Differences in amide shifts given by ΔδNHs.

The presence of β-structure in the cLID_567–592_ correlated with a localized high aggregation propensity predicted by TANGO (LIAFY_573–577_) (Supplementary Fig. S3). To determine how to remove this aggregation propensity while preserving helicity and suppressing β-strands, we scanned different combinations of residues in silico. Based on this analysis, we chose the mutations I574 G, F576G, I586G, L588G in HM1 and HM2 (hereafter *4G variants*; Fig. 4a, Supplementary Fig. S3) and introduced them both in the cLID_567–592_ (cLID_567–592–4G_) and NHE1-LID_539-593_ (NHE1-LID_539-59-4G_).

The cLID_567-592-4G_ was highly soluble and showed a typical CD-profile of a mainly disordered peptide, which was completely insensitive towards the addition of liposomes (Fig. 4b). However, in cLID_567–592_ variants with individually mutated hydrophobic pairs, denoted cLID_567-592-2G-1_ (I574G, F576G) and cLID_567-592-2G-2_ (I586G, L588G), addition of anionic liposomes induced a shoulder at 222 nm for both peptides (Fig. 4b) meaning some helix formation, and implying that these four residues are essential for, and in concert drive, cLID_567–592_ interaction with the membrane. The NHE1-LID_539-593-4G_ was expectedly disordered in the absence of a membrane mimetic and showed good solubility in buffer. In the presence of membrane mimetics, anionic lipids still induced helicity as judged by CD analyses, but at much lower amplitude than for wild-type NHE1-LID_539-593_ (Fig. 4c), compatible with helix formation only in the N-terminal region upon membrane association. Furthermore, the θ_222nm_/θ_208nm_ ratio was distinctly below unity in contrast to what is expected for a coiled-coil structure. NMR spectroscopy of NHE1-LID_539-593-4G_ confirmed this observation and showed that in the absence of a membrane mimetic, transient helicity was completely lost for residues in the C-terminal, with no large effects in the N-terminal (Fig. 4d, e). Adding LPPG to the NMR sample of NHE1-LID_539-593-4G_ further substantiated this conclusion, as under these conditions, and at low temperatures to enhance signal to noise, signals from the C-terminal region remained compatible with a disordered, non-membrane bound state (Supplementary Fig. S4).

Collectively, these results show that the non-amphipathic character of the cLID_567–592_ with hydrophobic side chains of I574, F576, I586, and L588 drives the membrane interaction of this part of the NHE1-LID and that the CD signature of a coiled-coil in the presence of membrane mimetics relies on these hydrophobic residues. In contrast, the N-terminal part of the NHE1-LID_539-593_, i.e. the nLID_542-569,_ associates electrostatically with the negatively charged membrane surface independently of the C-terminal part.

### The NHE1-LID_539-593_ is constitutively membrane-bound and embeds into the lipid head group layer

Because of the observed indications of coiled-coil αα-hairpin formation by CD analysis using bicelles, we sought to obtain direct insight into the NHE1-LID_539-593_ interaction using a more native-like membrane model system in the form of a supported lipid bilayer through Quartz-Crystal Microbalance with Dissipation monitoring (QCM-D)^45^ and Neutron Reflectometry (NR)^46^ experiments. By these methods we could characterize: i) NHE1-LID_539-593_ adsorption to a more native-like lipid bilayer (QCM-D and NR), ii) the overall structure of the NHE1-LID_539-593_ when bound to this bilayer (NR), and iii) the impact of NHE1-LID_539-593_ on the membrane structure (NR). Supported lipid bilayers composed of POPC and POPS (70 mol%:30 mol%) were used for both the QCM-D and NR experiments.

**Fig. 5:**
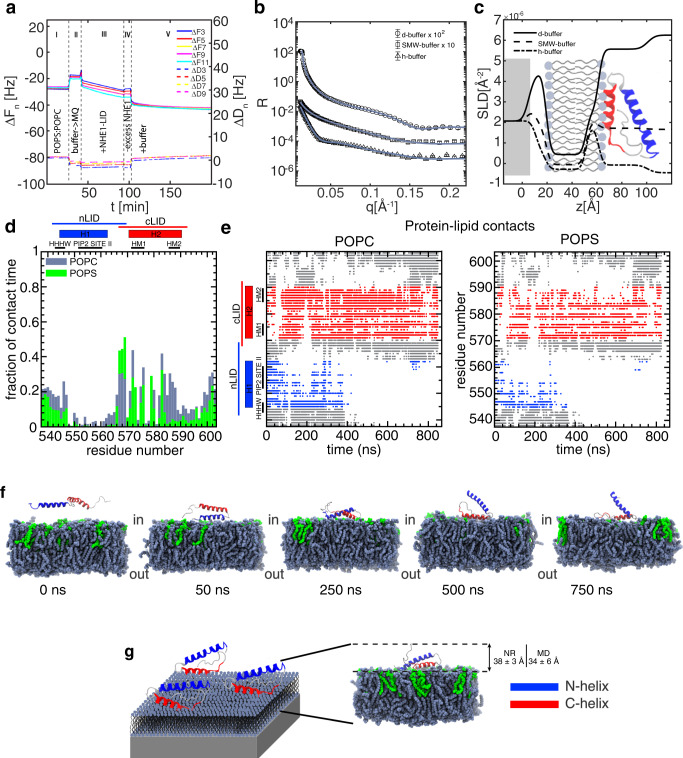
The membrane-bound structure of NHE1-LID. **a** QCM-D, with sensor frequency shift (ΔF) on the left y-axis (top lines) and the dissipation factor (ΔD) on the right y-axis (lower lines), colors represent different sensor harmonics reporting on the adsorption of molecules (i.e., lipids and subsequently NHE1-LID) on the sensor surface. Area I to V correspond to the supported lipid bilayer in contact with the sensor (I), injection of MQ water prior (II), injection of the protein solution and its incubation with the membrane (III), removal of excess protein with MQ water (IV) and re-introduction of the buffer (V). **b** Reflectivity vs. q measured in buffers of different degree of deuteration (see C). **c** Scattering length density (SLD) profiles obtained from the NR experiment; the experimental data were collected for the sample in contact with buffer prepared with different D_2_O content (d-buffer = 100% D_2_O, smw-buffer = 38% D_2_O:62% H_2_O, h-buffer = 100% H_2_O). **d** Per-residue histogram of protein-lipid contacts observed during the MD simulation (blue-gray: POPC, green: POPS). **e** Temporal evolution of protein-POPC and protein-POPS contacts from the MD simulation. **f** Snapshots from the MD simulation trajectory depicting the different bound orientations of NHE1-LID on the membrane. NHE1-LID shown in ribbon representation, lipids shown in van der Waals’ representation (hydrogens omitted for clarity), colors as in (D) (H1, blue; H2, red; POPC blue-gray; POPS, green). **g** Schematic representation of the NR experiment setup. Details on the right side depict the protein-layer thickness measured from NR compared to average thickness of the protein obtained from MD.

Initially, we monitored the interaction between the NHE1-LID_539-593_ and the supported lipid bilayer by QCM-D (Fig. 5a). In this method, the sensor frequency shift (ΔF) for the different sensor harmonics reports on the adsorption of molecules on the sensor surface, while the dissipation factor (ΔD) indirectly reports on the packing of molecules on the surface (Fig. 5a). ΔF and ΔD were monitored as a function of time (Fig. 5a). A decrease in ΔF with a corresponding increase in ΔD indicates an increase in adsorbed mass on the surface. The characteristic ΔF of ~ −25 Hz^47^ was first recorded for the POPC:POPS bilayer in buffer (area I, Fig. 5a). To avoid salt-induced precipitation of the NHE1-LID_539-593_, the buffer was replaced with MQ water (area II), the NHE1-LID_539-593_ was injected (area III) and after ∼30 min of incubation, excess protein was removed by MQ water (area IV) and buffer reintroduced (area V). By comparing region I and V, we observed a decrease in ΔF and increase in ΔD suggesting an increase in adsorbed mass (Fig. 5a). This shows that under these conditions, the NHE1-LID_539-593_ is adsorbed onto the POPC:POPS membrane and stays associated with it.

Similar experimental conditions were used to collect NR data. Initially, the structure of the lipid membrane was characterized (Supplementary Fig. S5a–c and Supplementary Table S1) and the data fitted to a three-layer model (head groups – acyl chains – head groups). Subsequently, the NR measurements were repeated upon injection of the NHE1-LID_539-593_ (Fig. 5b). The obtained scattering length density profile, *ρ*(z) (Fig. 5c) indicated how the different sample components, i.e. lipid head groups, lipid acyl chains, and the NHE1-LID_539-593_, were distributed in the direction normal to the support surface. The best model to describe the data consisted of four layers. Three layers described the lipid bilayer structure as detailed above. An additional layer was used to describe the NHE1-LID_539-593_ molecules and the membrane surface (protein – head groups). Inspection of the profile showed that the inner head group layer as well as the lipid acyl chain region, were unaffected by NHE1-LID_539-593_ adsorption (Supplementary Table S2). In contrast, the structure of the outer head group layer appeared affected by the presence of NHE1-LID_539-593_, suggesting NHE1-LID to be partially embedded here. The thickness of the protein layer on the membrane surface that produced the best fit to the NR data was 38 ± 3 Å (Fig. 5c). This is overall compatible with the expected thickness of NHE1-LID in an αα-hairpin structure with only one of the helices penetrating into the lipid layer. As a fully folded hairpin on the surface would be more compact (see below), this suggests that the other helix is lying dynamically on top of the first one. In addition, a fit assuming an extended helical conformation of the NHE1-LID along the surface of the membrane agrees poorly with the NR data (Supplementary Fig. S6), arguing against such a model.

Finally, we repeated the QCM-D and NR measurements for the nLID_542-569_ (Supplementary Fig. S7) and NHE1-LID_539-593_-_4G_ (Supplementary Fig. S8, Supplementary Table S3). Alone, nLID_542-569_ still interacted with the membrane, in agreement with the CD data and the NMR data on the NHE1-LID_539-593-4G_. A less negative value of ΔF (~-38 Hz) compared to that of NHE1-LID_539-593_ (~ −42 Hz) was observed. This could be explained by the lower molecular weight of nLID_542-569_ as compared to the NHE1-LID_539-593_, but could also reflect that a lower number of molecules were interacting with the membrane. Importantly, for NHE1-LID_539-593-4G_, the NR measurements suggested that the structure of the protein layer on the membrane surface differed from that of NHE1-LID_539-593_ (Supplemenatry Fig. S6), with NHE1-LID_539-593_ being located mainly outside the membrane without substantially affecting the lipid bilayer structure, in particular the outer headgroup layer. This, again, supports the observations made by CD and NMR spectroscopy.

Taken together, these results show that the NHE1-LID_539-593_ is partially embedded into the lipid head group layer and that the C-terminal region of the NHE1-LID_539-593_, with its overall hydrophobic character, inserts deeper into the membrane than the N-terminal region, which on its own, is associated on the membrane surface without penetration.

### A structural model of the NHE1-LID:membrane co-structure

To provide further details about the structure and dynamics of the membrane bound NHE1-LID_539-593_, we used molecular dynamics (MD) simulations. An atomistic model of the NHE1-LID was built covering residues C538-T603 and containing two predefined α-helices: H1 (H545-L562) and H2 (P571-E590). The helical regions were defined taking into account the NMR data both in the absence (Fig. 1d), and presence of a membrane mimetics (Fig. 2h; Supplementary Fig. S1), as well as secondary structure predictions (Supplementary Fig. S9a). A bilayer consisting of POPC in one layer and POPC:POPS (70 mol%:30 mol%) in the other, mimicking (and termed from here on) the outer and inner leaflet, respectively, of the plasma membrane was established, and the NHE1-LID538-603 model was placed near (~7 Å) the inner leaflet. The system was solvated and simulated for 860 ns. Analysis of the MD trajectory revealed that the NHE1-LID_538-603_ readily bound to the inner leaflet and remained bound for the duration of the simulation. Monitoring the frequency of the protein – lipid contacts (Fig. 5d) showed that three NHE1-LID regions mainly contributed to binding: (i) the most N-terminal part (C538-K547), (ii) the C-terminal part of the linker connecting H1 and H2 (mainly R567, S568 and K569), and (iii) several residues spanning the length of H2 (P571, Q572, A575, F576, K579, M582, K583, Q584, I586, E587, L588). Many of these latter residues (underlined) are part of the two hydrophobic motifs of H2. Plotting the time-evolution of the protein-lipid contacts (Fig. 5e) showed that most of the initial contacts were formed by the N-terminal H1 followed after 100 ns by several contacts by H2, most of which remained stable for the remainder of the simulation. During the second half of the simulation (t_sim_ > 400 ns), only residues from H2 and the inter-helix region had stable contacts with the inner leaflet. This suggests that the binding modes are dynamic and adaptable, consistent with the broad specificity indicated by the lipid-dot blots (Fig. 2a-d) and the lack of NMR signals in the 3D spectra in membrane mimetics. Snapshots of the trajectory are presented in Fig. 5f and Movie S1 (H1, blue – H2, red). The dynamics and preferred regions of contact to POPC and POPS were the same, except that that most of the protein-POPS contacts involved mainly positively charged residues (K569, K579, and K583), while a mixture of polar, charged and hydrophobic residues formed most of the contacts with POPC.

During the simulation, the NHE1-LID_538-603_ quickly adopted an αα-hairpin structure as shown by the immediate dramatic decrease in the angle between H1 and H2 (Supplementary Fig. S9b) and the abrupt increase in C^α^-RMSDs (Supplementary Fig. S9c) and also readily seen in the time course movie (Supplementary Movie S1). A series of structural accommodations followed, including shortening of H2 on its C-terminal end from E590 to Q584 (Supplementary Fig. S9d), in agreement with the SCSs observed by NMR (Fig. 4e). The αα-hairpin was stabilized mainly by contacts between residues from the C-terminal half of H1 and residues from the N-terminal half of H2 (Supplementary Fig. S9e). Interestingly, some of these H1-residues constitute the PI(4,5)P_2_ binding site II (K556, K560) while some residues from H2 belong to the hydrophobic LIAFY motif (L573, I574, Y577). The remaining two residues of this motif (A575, F576) contributed to membrane binding (Supplementary Fig. S9e).

To estimate the extent of NHE1-LID_538-603_ penetration into the lipid head group layer, allowing a more quantitative comparison of the MD and NR results, normalized averaged density profiles were obtained from the simulation, omitting the first 100 ns (S9f Fig). An overlap between the protein density and the densities of the POPC:POPS headgroups indicated some degree of penetration as expected from the protein-lipid contact measurements. No deeper penetration was observed, in agreement with the NR results. The average thickness of the protein on the surface of the membrane during the simulation was estimated to 34 ± 6 Å, in good agreement with the thickness of 38 ± 3 Å obtained by NR (Fig. 5g). In contrast, a linear, extended model of NHE1-LID_538-603_ with both helices fully in contact with the membrane would have a much smaller thickness of ~15 Å (Supplementary Fig. S6). These estimations suggest that the simulation captures the essential compactness of the NHE1-LID_538-603_ measured experimentally on the surface.

Taken together, and in line with CD data and NR analyses, MD simulations indicate that the NHE1-LID_538-603_ binds to an anionic lipid surface, forming an αα-hairpin structure of two helices, H1 and H2. The binding and the hairpin configuration are dynamic, with the most favorable and long-term stable contacts to the membrane made by the hydrophobic C-terminal residues of H2 (Fig. 5f). The amphipathic N-terminal H1 of the NHE1-LID_538-603_ forms the initial contacts with the bilayer and the NHE1-LID_538-603_ structure penetrates the outer headgroup layer of the lipid bilayer.

To address how the presence of PI(4,5)P_2_ in the membrane would affect the structure of the membrane-bound NHE1-LID, we performed a similar MD simulation placing the NHE1-LID model near a POPC:PI(4,5)P_2_ (80 mol%:20 mol%) membrane. As for the POPC:POPS (70 mol%:30 mol%) membrane, we observed binding of the protein to the bilayer and formation of a helix-helix hairpin. However, in the presence of PI(4,5)P_2_, the pattern of contacts between the NHE1-LID and the membrane was different: In this case, most of the contacts with the lipid were stablished by residues from H1, and highly specific interactions were observed between residues known to form the PI(4,5)P_2_ binding site II (R552, K555 and K556) (Supplementary Fig. S10a, b). Thus, lipid composition affects both the distribution of membrane-bound states of the NHE1-LID and the dynamics of the bound state. Furthermore, we observed that the secondary structure of NHE1-LID in the POPC:PI(4,5)P_2_ (80 mol%:20 mol%) membrane evolved differently over the simulation as compared to the POPC:POPS (70 mol%:30 mol%) system. With POPC:PI(4,5)P_2,_ we observed a shortening of H1 due to unfolding and penetration into the membrane of the first seven N-terminal residues and a braking of H2 into two smaller helices, keeping the overall helical content similar (Supplementary Fig. S10c, d). This also matches the observation of no detectable difference in helical content by CD (Fig. 3b). Accordingly, the thickness of the protein outside the bilayer was reduced to ~20 Å. Additionally, we observed that during the simulation, PI(4,5)P_2_ accumulated around NHE1-LID as shown in the average density maps (Supplementary Fig. S10e–g), which explains the long lived contacts between residues from H1 and these lipids.

These results show that the structure and dynamics of NHE1-LID are affected differently by the presence of POPS or PI(4,5)P_2_ in the bilayer. The observed change in protein-lipid contact profiles indicates that the NHE1-LID may respond to changes in the membrane composition and change orientation or conformation upon binding to different charged lipids.

### The overall folded structure of the NHE1-LID is essential for exchanger activity

**Fig. 6:**
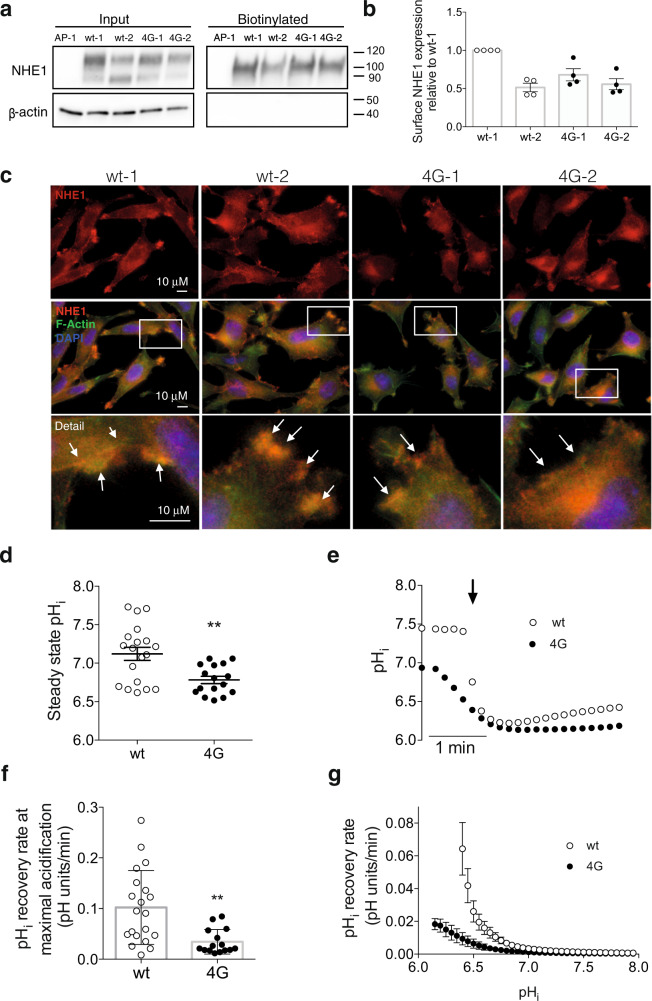
NHE1 activity, but not membrane localization, is attenuated by disruption of the NHE1-LID:membrane costructure. **a**, **b** Plasma membrane expression of NHE1 in AP-1 cells (untransfected or stably expressing wt or 4G-NHE1 as indicated), assessed by pull-down of the biotinylated membrane fraction followed by immunoblotting for NHE1. **a** Representative immunoblots. β-actin was used as a loading control (no signal in the pull-down fraction, as expected). Uncropped blots are available in Supplementary Fig. S12. **b** Plasma membrane NHE1 expression normalized to that in wt clone 1. Data are quantified from n = 4 biologically independent experiments per clone and shown as mean with S.E.M error bars and all individual data points. The membrane expression of 4G-NHE1 did not differ significantly from that of NHE1 (pooled data from both clones for each variant, two-tailed, non-paired Student’s *t*-test, *p* > 0.05). **c** Localization of wt- and 4G-NHE1 in AP-1 cells. Cells were fixed and stained with antibody against NHE1 (red), rhodamine-conjugated phalloidin to visualize F-actin (green), and DAPI to visualize nuclei (blue). Arrows in the detail images highlight the membrane localization of NHE1. Data shown are representative of *n* = 3 biologically independent experiments per condition. **d**–**g** To measure wt- and 4G-NHE1 activity, cells were loaded with BCECF-AM, and pH_i_ monitored using real time fluorescence imaging. **d** Steady-state pH_i_, averaged over the two NHE1 wt (*n* = 19 biologically independent experiments) and 4 G (*n* = 16 biologically independent experiments) cell clones. Data are shown as mean with S.E.M. error bars and all individual data points. **e** Representative examples of pH_i_ traces. The black arrow indicates the point of removal of NH_4_Cl. **f** pH_i_ recovery rates for the NHE1 wt (*n* = 20 biologically independent experiments) and 4 G (*n* = 15 biologically independent experiments) variant, calculated from the initial linear part of the pH_i_ traces after maximal acidification, as in **e**. Data are shown as mean with S.E.M. error bars. **g** From traces as in (E), pH_i_ recovery rates as a function of pH_i_ was calculated by fitting the recovery rates over the entire recovery period. Data are shown as mean with S.E.M. error bars. Data in F and G were corrected for relative cell surface expression (data from Panel B) to ensure that the pH_i_ recovery represents the capacity of the membrane-expressed fraction of NHE1. **p = 0.0024 (panel D) and 0.0016 (panel F) and compared to wt, two-tailed, non-paired Student’s *t*-test using GraphPad Prism 8.4.1 software and assuming normal distribution. Source files for Fig. 6b,d–g available as supplementary data.

To understand the role of the NHE1-LID membrane co-structure in NHE1 transport activity, we generated a full length NHE1 with the LID_4G_-mutations and expressed this and the wild-type (wt) NHE1 in AP-1 cells - mammalian epithelial cells lacking endogenous NHE activity^48^. In the absence of HCO_3_^−^, native AP-1 cells have no pH_i_ recovery capacity and hence all pH_i_ recovery can be attributed to the exogenously expressed NHE1^48,49^. To ensure that the observed effects were not due to clonal variation, two stable AP-1 cell clones expressing wt-NHE1, and two stable clones expressing the 4 G variant NHE1 (4G-NHE1) were generated and functionally investigated. While there was some clone-to-clone variation in expression as assessed by Western blot analysis, overall plasma membrane localization of NHE1 was not compromised by the 4G-mutations, as seen by the comparable NHE1 band intensity in the biotinylated fraction for all variants (Fig. 6a,b; Supplementary Fig. S12). Consistent with this, immunofluorescence analysis indicated a similar plasma membrane localization of wt- and 4G-NHE1 in AP-1 cells (Fig. 6c). Allosteric activation of NHE1 is dependent on dimer formation^50^, which could potentially be altered by mutations in the LID region. However, Western blots to detect the existence of NHE1 dimers in cell lysates did not indicate detectable differences in dimer formation between wt- and 4G variants under these conditions (Supplementary Fig. S11), consistent with previous results indicating the involvement of other regions in NHE1 in overall dimer formation^51,52^. Figure 6d shows that steady state pH_i_ in the absence of HCO_3_ was significantly lower in cells expressing the 4G variant compared to cells expressing wt NHE1. To determine whether this reflected altered NHE1 activity, cells were exposed to an NH_4_Cl prepulse followed by NH_4_Cl removal to induce intracellular acidification^53^, eliciting a phase of recovery of pH_i_, all in the absence of HCO_3_^−^. Recovery rates were averaged over the two cell clones for each condition, and pH_i_ recovery was furthermore normalized to NHE1 surface expression to ensure that recovery rates reflect regulation of activity rather than possible differences in expression. Figure 6e shows representative traces of pH_i_ over time, panels F and G show the pH_i_ recovery rates at the time of maximal acidification (Fig. 6f) and as a function of pH_i_ (Fig. 6g). Remarkably, in cells expressing the 4G-NHE1, steady state pH_i_ was reduced from about 7.2 to about 6.8 (Fig. 6d), and the rate of pH_i_ recovery from acidification was reduced by about 80% compared to that of cells expressing wt NHE1 (Fig. 6e-g). The reduced recovery rate for the 4G-NHE1 variant was seen across all pH values, and the set-point for detectable NHE1 activation was shifted to more acidic pH_i_ values (Fig. 6g).

Collectively, these results demonstrate that the 4G mutations disrupting the NHE1-LID-membrane co-structure strongly reduce NHE1 transport activity without detectably affecting NHE1 membrane localization.

## Discussion

Unraveling the dynamics in membrane proteins is essential for understanding their functions^54^. While dynamics in folded regions can be determined and monitored using simulations and experiments, a largely overlooked part of membrane proteins come from their disordered regions, which are often removed for structural studies or are neglected in models. Here, we show the key importance of membrane interactions with a disordered region of the ubiquitous Na^+^/H^+^ exchanger NHE1. We propose that such interactions are likely to play equally important roles in many other membrane proteins, as many as 30% of which have been estimated to contain disordered tail regions^1^.

It is firmly established that the proximal part of the C-terminal tail of NHE1 interacts with the plasma membrane^26,27,31,32^, yet a structural understanding of this interaction has been lacking. In the absence of a membrane, the NHE1-LID is intrinsically disordered. A key finding of the present work is that upon contact with anionic lipids, the NHE1-LID forms a structure consisting of two α-helices in an αα-hairpin structure, the most C-terminal of which anchors the NHE1-LID to the membrane. In contrast, in the absence of a membrane, the NHE1-LID is intrinsically disordered and highly sensitive to environmental changes. We show that disruption of this co-structure between the membrane and the two LID helices is associated with a profound reduction in cellular NHE1 activity.

Our MD simulation showed that the NHE1-LID formed an αα-hairpin structure on the membrane. Both helices formed by the LID (H1 and H2) interact with the membrane bilayer, but H2 makes more stable contacts to the lipid head-group layer. Consistent with the importance of H2 in membrane interaction, NR data showed that NHE1-LID penetrates less deeply into the lipid headgroup layer when H2 is disrupted and less hydrophobic (the NHE1-LID_4G_ variant). Given the extreme aggregation properties of H2 on its own, and its loss of membrane interaction when mutating hydrophobic residues, this region of NHE1-LID is suggested to be constitutively in contact with, and partially buried by, the membrane, acting as a tether for membrane interaction of the NHE1 C-tail. In contrast, H1, whether studied alone in the form of the nLID_542-569_ peptide or by mutating H2 (NHE1-LID_4G_), interacts electrostatically with the membrane without visible penetration into the head-group layer, as seen by NR, and forms a membrane-induced amphipathic helix independent of H2 as shown by CD and NMR. The thickness of the protein layer on the membrane as measured by NR and recapitulated by the MD, suggests that within the αα-hairpin structure, H1 partially overlays H2 with contacts to the membrane mainly involving residues in its N-terminal end. In this protein:membrane co-structure, the hydrophobic motifs of H2, LIAFY_572-577_ and AIELV_585-589_ are essential, both because they stabilize the αα-hairpin structure, and because they interact directly with the membrane. Indeed, it appears from the MD simulation that the first motif, LIAFY_572-577_, forms inter-helical contacts to H1 as well as with the membrane, whereas the second hydrophobic motif, AIELV_585-589,_ forms more contacts with the membrane. Furthermore, the aromatic residues of the LIAFY_572-577_-motif, either alone or in combination with W546 of H1, may additionally stabilize the co-structure by engaging in one or more T-stacks. Interestingly, a similar hydrophobic cluster (VNDSILFL) was recently identified in the hydrophobic kinase associated-1 domain of certain kinases, where it serves as a membrane-anchor surrounded by basic residues^55^, suggesting hydrophobic anchors as broadly used features in proteins. Both the LIAFY_572-577_ and AIELV_585-589_ motifs also bear similarity to hydrophobic, short membrane insertion loops reported in PX and FYVE domains, which typically have aromatics and/or valine or isoleucine residues involved in membrane binding^56,57^. Thus, the hydrophobic region of the NHE1-LID may serve as a membrane tether, whereas the basic clusters contribute to the membrane interaction by electrostatic interactions with anionic phospholipids. Importantly, our data suggests that the intrinsically disordered LID of NHE1 must go through a folding-upon-binding process forming a co-structure with the membrane. This is highly similar to what has been observed for many IDPs that fold-upon-binding to protein partners^58–60^ and to membranes, exemplified, e.g., by α-synuclein^4,61,62^. The bipartite exploitation of both hydrophobic and electrostatic interactions is also similar to interactions reported for other proteins with membrane association^7,63–65^. Moreover, changing the lipid composition of the membrane or changing pH modulated the structure and dynamics of the NHE1-LID without loss of binding, suggesting it to be responsive to the membrane lipid composition, likely as a result of its basic disordered nature. We propose that this has direct implications for regulation of NHE1 upon changes in cellular lipid signaling, e.g. during phospholipase activation by G-protein coupled receptors and receptor tyrosine kinases, leading to changes in membrane PI(4,5)P_2_ content.

The extensive restructuring of NHE1-LID organization on the membrane caused by the 4G mutations of the hydrophobic H2, also lead to a profound reduction of NHE1 transport activity after an acid load compared to that of wt NHE1, when the transporters were expressed in mammalian cells lacking endogenous NHE activity. This was not associated with a detectable loss of overall localization of NHE1 to the membrane, suggesting that this region is not essential for NHE1 membrane localization but rather modulates NHE1 activity. The loss of integrity of the membrane:protein co-structure was associated with an acidic shift in the pH_i_ required for detectable NHE1 activity as well as a reduced steady state pH_i_ under HCO_3_^−^ free conditions, where the exogenously expressed NHE1 is the only detectable mechanism of pH_i_ regulation in these cells. This supports previous work pointing to a role of NHE1-lipid interaction in pH sensing^26,32^, and provides the first structural insights into how the NHE1-LID interacts with the membrane and directly demonstrates the functional importance of this interaction.

Interaction with lipids, and in particular anionic phospholipids, is essential for full NHE1 activity^26,27,32^. Mutation of I574 and F576 in the hydrophobic LIAFY_573-577_-motif abolished both LID instability in the absence of lipids and LID membrane binding, and the additional mutations, I586G and L588G abolished LID aggregation propensity in the absence of lipids as well as membrane binding of the cLID_567-592_. Finally, the mutations eliminated helicity in the C-terminal region of NHE1-LID. Although we cannot fully rule out that the 4G mutations also interfere with an interaction between NHE1 and a yet-to-be-described protein binding partner in this region, we favor the interpretation that these long, hydrophobic residues are important for the constitutive contact of the NHE1-LID with the membrane. This is likely both through direct and stable interaction with the membrane and through stabilizing the αα-hairpin structure. With H2 penetrating into the lipid head-group layer, this also makes the NHE1-LID potentially relevant in terms of sensing of changes in membrane curvature, e.g. during osmotic shrinkage and mechanical stimuli such as cell spreading, which are well known to regulate NHE1^12^. Notably, such a role is likely to depend on the cLID_567-592_, as deletion of the PI(4,5)P_2_ and ERM binding sites in the nLID_542-569_ did not alter NHE1 mechano-sensitivity^66^.

Several protein domain types interact with membranes in a pH-dependent manner due to histidine (de)protonation, as shown for FYVE^67^, PH^68^ and ENTH^69^ domains. Similarly, the N-terminal part of the LID region of NHE1 has previously been implicated in pH sensing: a histidine cluster (^540^HYGHHH^545^), partially overlapping with H1 as identified here and conserved in NHE1 across species, was shown to be involved in, but not sufficient for, NHE1 pH sensitivity^32^; in good agreement with several previous reports of other modes of NHE1 pH responsiveness^12^. We did not observe changes that suggested release of the NHE1*ct*, NHE1-LID, or nLID_542-569_ from the membrane bound state upon changes in pH in vitro, and the helical content of H1 was not altered by pH changes in the physiological range. Thus, while the work by Webb and colleagues (2016) clearly demonstrates that the histidine cluster possesses pH sensitivity, our data indicate that the membrane association of the NHE1-LID is not a binary on-off switch in which the NHE1-LID becomes fully released from the membrane with changes in pH_i_. Instead the dynamics of the LID on the membrane indicates a structure that can be allosterically modulated.

Many membrane proteins contain IDRs of considerable length^1^ which typically play roles in scaffolding of signaling and adapter proteins, as well as in binding to folded domains that can act as membrane curvature sensors^70^. Furthermore, many kinases that are actively involved in signaling have been shown to bind to disordered regions, and can themselves either be anchored to the membrane through modifications by acylation^71–73^ or engage in lipid interactions via designated lipid binding domains, such as a phosphoinositide binding site within the FERM domain of several tyrosine kinases^74–76^ or within SH2 domains of Src kinases^77,78^. For NHE1, the LID as a disordered region takes on these roles directly, acting as an intrinsic membrane anchor. We propose that the NHE1-LID acts as a coincidence detector sensitive to the combination of multiple conditions, such as pH and lipid composition, or pH and the presence of other binding partners or membrane curvature and ionic strength. To our knowledge, this is a novel mechanism of membrane protein regulation by structural disorder. It is noteworthy that the residues forming the second PI(4,5)P_2_ binding motif (R552 – K560) do not establish stable contacts with lipids during our MD simulation, but rather participate in the H1–H2 interface. Binding of PI(4,5)P_2_ or the presence of other types of lipids or ATP interacting with different subsets of residues could potentially directly modulate the H1–H2 orientation and thus the conformation of the co-structure. Several intriguing questions remain, including by which mechanisms and factors such conformational modulation may occur, the number of possible states, and the extent of the secondary and tertiary structural changes. Finally, it is also possible that the NHE1-LID membrane co-structure forms a scaffold onto which other regions of the NHE1 tail – with or without bound interaction partners - may fold or bind, leading to the formation of higher-order complexes. The dynamics within these complexes may be dependent on the membrane lipid composition, as evident when comparing the behavior of the NHE1-LID when bound to membranes of different compositions.

In conclusion, we have identified and structurally described a co-structure formed between the NHE1-LID and the membrane. In this structure, the NHE1-LID forms an αα-hairpin structure in which a C-terminal helix, H2, penetrates the lipid head groups and anchors the NHE1 tail to the membrane using long hydrophobic side chains. The N-terminal helix, H1, interacts electrostatically with anionic lipids, forming contacts C-terminally with the N-terminal part of H2. When this co-structure is compromised by mutations in the C-terminal hydrophobic motifs, cellular NHE1 activity is severely impaired. Finally, we suggest that the conformation of the co-structure is dynamic and its structure sensitive to multiple physico-chemical cues. Precisely how such environmental changes manifest in structural transformations within the co-structure remains to be elucidated. We suggest that similar mechanisms may be operational in the IDRs existing in many other intracellular domains of membrane proteins.

## Methods

### Protein expression and purification

H_6_-CHP1 was co-expressed with S-tagged NHE1 V503-G595, or V503-R698 (C561A), from a pET-Duet-vector. A 10 mL overnight (o/n) culture of freshly transformed *E. coli* BL21 CodonPlus DE3-RP was transferred to 1 L LB media containing ampicillin (amp) and grown to OD_600_ of ~0.6 before induction with 0.5 mM isopropyl-β-D-1-thiogalactopyraoside (IPTG). Proteins were expressed for 3 h at 37 °C and 160 rpm. Cells were harvested by centrifugation, the cell pellet suspended in 50 mL sonication buffer (10 mM Hepes/Tris pH 8.0, 6 M GdnHCl, 0.5 M NaCl), and cells broken by sonication on ice. The cell lysate was cleared from debris by centrifugation, mixed with 4 mL Ni-NTA resin slurry and incubated on ice for 15 min. The slurry was transferred to a column, washed with 20 mL sonication buffer, followed by two times 20 mL refolding buffer (10 mM Hepes/Tris pH 8.0, 75 mM imidazole, 0.5 M NaCl, 0.1 mM CaCl_2_). The protein complex was eluted with 6 mL elution buffer (10 mM Hepes/Tris pH 8.0, 500 mM imidazole, 100 mM NaCl, 0.1 mM CaCl_2_). Protein containing fractions were loaded onto a 24 mL Superdex 200 10/30 GL (GE Healthcare) that was equilibrated with 20 mM TrisHCl pH 8.0, 150 mM NaCl, 0.1 mM CaCl_2_. Protein containing fractions were pooled. All protein preparations were >95% pure judged from SDS-PAGE.

A pGEX vector coding for GST-tagged NHE1-LID or NHE1-LID_4G_ including a thrombin cleavage site was transformed into *E. coli* BL21 CodonPlus DE3-RP cells and grown o/n at 37 °C in 10 mL LB media with 100 μg/mL amp and 35 μg/mL chloramphenicol (cam). For expression of un-labeled NHE1-LID and NHE1-LID_4G_, the cultures were added to 1 L LB media with 100 μg/mL amp and 35 μg/mL cam and grown at 37 °C, 185 rpm to an OD_600_ of 0.6–0.8 and induced with 0.5 mM IPTG. For expression of stable isotope labeled NHE1-LID and NHE1-LID_4G_, the o/n culture was added to 1 L LB media with 100 μg/mL amp and 35 μg/mL cam and grown at 37 °C, 185 rpm to an OD_600_ of 0.6–0.8. The cells were gently pelleted at 4000 g for 10 min at 4 °C and transferred to 400 mL M9 minimal media (22 mM KH_2_PO_4_, 42 mM Na_2_HPO_4_·2H_2_O, 17 mM NaCl, 1 mM MgSO_4_) added 1:1000 of M2 trace element solution, 20 mM glucose (if required: ^13^C-labeled), 19 mM NH_4_Cl (if required: ^15^N-labeled) with 100 μg/mL amp and 35 μg/mL cam. Cells were incubated at 37 °C, 185 rpm for 30 min before induction with 0.5 mM IPTG and harvested after 3 h by centrifugation at 4,700 g for 40 min at 4 °C. After resuspension in 25 mL 50 mM Tris, pH 7.0 and centrifugation at 4700 g for 40 min at 4 °C, the pellet was dissolved in 20 mL lysis buffer (50 mM Tris, 2.5 mM CaCl_2_, 0.5 mM MgCl_2_, ethylenediaminetetraacetic acid (EDTA)-free protease inhibitor (Roche), pH 7.4), 200 μg/mL lysozyme was added (Sigma), followed by 40 min incubation at RT under gentle shaking. DNAse I (AppliChem) was added to a final concentration of 20 µg/mL and incubated for 20 min at 37 °C under gentle shaking before cells were lysed by French press (American Instrument Company). The sample was then centrifuged at 15,000 g for 30 min at 4 °C. The lysate pellet was resuspended in 10 mL wash buffer I (50 mM Tris, 1 mM EDTA, 1 mM β-mercaptoethanol, 2% Triton X-100, pH 7.4), centrifuged, the supernatant was discarded and the wash repeated. The pellet was resuspended in 10 mL wash buffer II (50 mM Tris, 1 mM β-mercaptoethanol, pH 7.4), centrifuged and the supernatant discarded. The pellet was then dissolved in 10 mL phosphate-buffered saline (PBS) (137 mM NaCl, 2.7 mM KCl, 10 mM Na_2_HPO_4_, 1.8 mM KH_2_PO_4_), 1.5% (w/v) sodium lauroyl sarcosinate (sarkosyl), 1 mM dithiothretiol (DTT), pH 7.2 and dialyzed against 3 L PBS, 0.5% (w/v) sarkosyl, pH 7.2 in a 6–8 kDa cut-off Spectra/Por® dialysis tube (Spectrum Labs) at 4 °C o/n. The GST-NHE1-LID fusion protein was cleaved by 30 U thrombin (SERVA) for 24 h at RT under rotation and further purified by hydrophobic interaction chromatography on a 5 mL HiTrap Butyl HP column (GE Healthcare) washed with 5 column volumes (CV) of MQ water, followed by 5 CV 50 mM NaH_2_PO_4_/Na_2_HPO_4_, pH 7.0 (Buffer HB). A gradient from 0 to 100% 50 mM NaH_2_PO_4_/Na_2_HPO_4_, 1 M (NH_4_)_2_SO_4_, pH 7.0 (Buffer HA) over 5 CV, continued by washing with 5 CV 100% buffer HA. Prior to loading, 10 mM DDT was added to the sample, and a gradient from 50–100% buffer HB over 2.5 CV, was applied. Fractions containing NHE1-LID were pooled and lyophilized o/n. Subsequently, the sample was applied on a Zorbax 300StableBond C18 5 μm 9.5/250 column (Agilent) washed with 5 CV of 20% (v/v) ethanol, followed by 5 CV of 0.1% (v/v) trifluoroacetic acid (TFA) (Buffer RA). The pooled fractions were dissolved in 1 mL buffer RA, 20 mM DTT was added and a gradient of 0–100% 0.1% TFA, 70% (v/v) acetonitrile (Buffer RB) over 10 CV with a flow rate of 2.5 mL/min applied. Fractions containing LID were pooled, lyophilized o/n and stored at −20 °C. All protein preparations were >95% pure as judged from SDS-PAGE.

GST-tagged NHE1-LID_4G_ fusion protein was purified by affinity chromatography by loading the lysate supernatant on to a 2.5 mL Gluthathione Sepharose 4B column (GE Healthcare) and incubated for 1.5 h, at RT under slow rotation prior to being washed with 30 mL PBS, pH 7.3 (Binding Buffer). The GST-NHE1-LID_4G_ was eluted using 20 mL 50 mM Tris, 10 mM reduced glutathione (Sigma), 10 mM DTT, EDTA-free protease inhibitor (Roche), pH 8.0 (Elution Buffer). The sample containing GST-NHE1-LID_4G_ was cycled onto the column for a total of three times. The fusion protein was cleaved by 2.5 U of thrombin pr. mg GST-NHE1-LID_4G_ at 4 °C o/n, followed by heating of the sample to 72 °C for 5 min. The precipitate was pelleted by centrifugation at 20,000 g for 20 min at room temperature and discarded and the supernatant applied on a 1.66 mL μRPC C2/C18 column (GE Healthcare) washed with 5 CV of buffer RA. TFA was added to the sample at a final concentration of 0.1% (v/v) and 20 mM DTT prior to loading and a gradient of 0–100% buffer RB over 10 CV with a flow rate of 1 mL/min applied. Fractions containing NHE1-LID_4G_ were pooled, lyophilized o/n and stored at −20 °C. All protein preparations were >95% pure judged from SDS-PAGE.

Peptides covering the N-terminal and C-terminal parts of LID were purchased from Schafer-N, 5 mg and >95% pure. N-terminal LID (N-terminal lipid binding region of NHE1): Ac-GHHHWKDKLNRFNKKYVKKCLIAGERSK-NH_2.542-569_. C-terminal LID (C-terminal Lipid binding region of NHE1): Ac-RSKEPQLIAFYHKMEMKQAIELVESG-NH_2.567-592_ cLID^IAF-GAG^: Ac-RSKEPQLGAGYHKMEMKQAIELVESG-NH_2.567-592_.

### Lipid overlay assay

Pre-dotted lipid dot plots were purchased from Echelon (P-6001). All incubating/washing steps were done under gentle agitation. The membranes were blocked with TBS buffer (10 mM Tris, 150 mM NaCl, pH 7.2 or 8.2) supplemented with 1% (w/v) delipidated BSA (Sigma) for 1 h at room temperature. Next, 10 µg/mL protein was added in 5 mL TBS buffer supplemented with 1% (w/v) delipidated BSA and incubated o/n at 4 °C. All following steps were done at room temperature. The membrane was washed 3 times with 5 mL TBS supplemented with 0.1% (v/v) Tween-20 for 10 min each. The primary mouse monoclonal antibody against the S-tag (Novagen) was added 1:5000 in blocking buffer and incubated for 1 h, follwed by three washing steps as above. The secondary antibody was HRP coupled monoclonal against mouse, added 1:2000 in blocking buffer and incubated for 1 h, followed again by 3 washing steps. Bound protein was detected by chemiluminescence. Images were aquired with an Image Quant LAS-4000 with a CCD camera system.

### Preparation of liposomes

Lipids (1-palmitoyl-2-oleoyl-glycero-3-phosphocholine (POPC), 1-palmitoyl-2-oleoyl-glycero-3-phosphoserine (POPS), and 1,2-di-palmitoyl-phosphatidylinositol 4,5-diphosphate

(PI(4,5)P_2_) were purchased from Avanti Polar Lipids or Sigma Aldrich. The lipids were weighed out and dissolved in 1:1 methanol/chloroform. The solvent was evaporated using nitrogen-gas for approximately 30 min, creating a thin lipid film. The lipids were lyophilized o/n and stored at 4 °C or at −20 °C, until further preparation. The lipid films were hydrated in 20 mM sodium phosphate buffer pH 7.2, resulting again in a total lipid concentration of 25 mM. SUVs used in experiments with salt were dissolved in the respective buffer with 150 mM NaF. The lipids were put on ultrasound bath for approximately 6 h, followed by sonication using a UP400S Ultrasonic sonicator, 100% amplitude, in rounds of 5 s, until the solution turned clear - indicating the formation of SUVs. SUVs were stored at 4 °C for no longer than a week. Liposomes including PI(4,5)P_2_ were used within 24 h.

### Preparation of bicelles and micelles

DMPG (1,2-dimyristoyl-sn-glycero-3-phosphorylglycerol, Avanti) in chloroform was aspirated, transferred to a glass tube and placed under a nitrogen-gas flow until a lipid film had formed and subsequently lyophilized o/n to be dissolved in MQ. DMPC (1,2-dimyristoyl-sn-glycero-3-phosphocholine, Avanti) and DHPC (1,2-dihexanoyl-sn-glycero-3-phosphocholine, Avanti) were weighed out from powder stocks and dissolved in MQ. Lipid stock in MQ were mixed to obtain a lipid:detergent ratio (q-value) of 0.5 for both DMPC:DHPC and DMPG:DMPC:DHPC bicelles and additionally a DMPG:DMPC ratio of 1:2.33 (mol:mol). The bicelle stocks were then subjected to four cycles of vortexing, 45 s flash freezing in liquid nitrogen and 5 min heating at 45 °C. DHPC and LPPG (1-palmitoyl-2-hydroxy-sn-glycero-3-[phospho-rac-(1-glycerol), Avanti) were weighed out from powder stocks. Both detergents were dissolved in MQ.

### Circular Dichroism (CD) spectropolarimetry

Far-UV CD measurements were done on a Jasco J-810 spectropolarimeter with Peltier control at 20–25 °C using a 1 mm path length, from 260 to 190 nm with a scan speed of 20–50 nm/min, 3–20 accumulations, and with a data pitch of 0.1–0.5 nm. Peptide samples were prepared in 20 mM borate buffer pH 7.4, in 20 mM NaH_2_PO_4_ pH 5.0, 6.0 or 8.0. Peptide concentrations were 30–50 μM. SUV/LUVs were dissolved in the respective buffers matching the pH (SUV/LUV A: 80 mol% POPC, 20 mol% POPS; SUV/LUV B: 79 mol% POPC, 20 mol% POPS, 1 mol% PI(4,5)P_2_ – and otherwise indicated; 100 mol% POPC, 20 mol% POPS, 80 mol% POPC, 5 mol% PI(4,5)P_2_, 95 mol% POPC, 5 mol% LPA, 95 mol% POPC) and concentrations were varied between 0–10 mM. NHE1-LID and NHE1-LID_4G_ samples were first prepared in MQ, and subsequently diluted into micelles and bicelles prepared as described for resulting in a concentration of 15 μM NHE1-LID or NHE1-LID_4G_, MQ, 0.045 mM DTT, pH 6.0 and 6.4, respectively. For CD samples of NHE1-LID and NHE1-LID_4G_ in the presence of 2% (w/v) DMPC:DHPC the pH was 5.9 and 5.1, respectively, and in the presence of 25 mM DHPC the pH was 5.8 and 5.9, respectively. In the presence of 2% (w/v) DMPG:DMPC:DHPC the pH was 5.7 and 5.9, respectively, and in the presence of 2% (w/v) LPPG the pH was 7.0 and 6.9, respectively. All spectra were buffer-corrected and smoothened by the Jasco Spectra Manager smoothening correction tool, with 5 nm convolution width. Mean residual ellipticity θ_MR_ was calculated using the equation:

1 $$\left[ {\uptheta} \right]_{MR} = \frac{{\uptheta }}{{10 \cdot c \cdot l \cdot n}}$$

where θ is the measured elipticities in degrees, c is protein concentration in molar, l is the cell path length in cm and n is the number of peptide bonds in the protein. Only data below high tension (HT) voltage of 600 V was included.

The population of helicity was calculated according to^79^, using the following equation:

2 $${\mathrm{\% }}_{Helix} = \frac{{100}}{{1 + \frac{{{\uptheta }}_{MR(222)} - {\uptheta}_{MR(Helix)}}{{{\uptheta }}_{MR(Coil)} - {\uptheta}_{MR(222)}}}}$$

where θ_*MR*(222)_ is the calculated mean residual ellipticity at 222 nm from Eq. (1) and θ_*MR*(*Helix*)_ and θ_*MR*(*Coil*)_ are calculated from the following Eqs. (3) and (4), respectively:

3 $${\uptheta}_{MR(Helix)} = - 39,500\left( {1 - \frac{{2.57}}{n}} \right) + 100t$$

4 $${\uptheta}_{MR(Coil)} = 400 - 45t$$

Where *n* is the number of amino acids and *t* is the temperature in °C.

### Fluorescence spectroscopy

Fluorescence emission measurements were performed on a Perkin–Elmer LS 50B instrument at RT using a 5 mm square cuvette. Intrinsic tryptophan emission spectra of nLID_542-569_ were recorded from 300–400 nm by excitation at 280 nm, 3 accumulations and ~4 mm slit-widths. Sample preparations were done as for CD spectroscopy. Fluorescence spectra were quantified by the center of spectral mass <λ > analysis:

5 $$\lambda {\mathrm{ = }}\frac{{{\sum} {\lambda _i \cdot {\boldsymbol{F}}_{\boldsymbol{i}}} }}{{{\sum} {{\boldsymbol{F}}_{\boldsymbol{i}}} }}$$

### NMR spectroscopy

All NMR spectra were acquired at 298.15 K unless otherwise stated, on a Bruker Ascend 600 MHZ (^1^H) spectrometer or a Varian Unity Inova 750 MHz (^1^H) equipped with Bruker cryo probes or a Varian Unity Inova 800 MHz (^1^H) spectrometer equipped with a room temperature prove. Multidimensional spectra were recorded using non-uniform sampling. Free induction decays were processed by referencing internally relative to 4,4-dimethyl-4-silapentane-1-sulfonic acid (DSS), transformed using qMDD^80^, visualized in NMRDraw (a component of NMRPipe^81^) and analyzed using CcpNMR Analysis^82^. Assignments of backbone nuclei were performed manually from analysis of ^1^H-^15^N HSQC, HNCACB, CBCA(CO)NH, HN(CA)CO and HNCO spectra. Purified ^15^N,^13^C- (or ^15^N)-stable isotope labeled NHE1-LID and NHE1-LID_4G_ were subjected to NMR experiments. Samples of 330 μL of 200 μM NHE1-LID, 20 mM borate buffer (boric acid-NaOH), 0.5 mm DSS, 10% (v/v) D_2_O, 2 mM DTT at pH 6.4, 120 μM NHE1-LID, MQ, 0.5 mM DSS, 10% (v/v) D_2_O, 2 mM DTT at pH 6.5 and 80 μM NHE1-LID_4G_ in MQ, 0.5 mM DSS, 10% (v/v) D_2_O, 2 mM DTT at pH 6.5 were prepared and transferred to Shigemi NMR tubes and recorded at 298.15 K. For investigation of LPPG micelle binding by NMR the NHE1-LID or NHE1-LID_4G_ was first dissolved in MQ and then diluted into LPPG micelles prepared as described, followed by addition of NaH_2_PO_4_/Na_2_HPO_4_ resulting in a concentration of 257 μM NHE1-LID in 20 mM NaH_2_PO_4_/Na_2_HPO_4_, 2% (w/v) LPPG, 0.5 mm DSS, 10% (v/v) D_2_O, 2 mM DTT at pH 6.8. For NHE1-LID_4G_ the final concentration was 72 μM. ^1^H-^15^N HSQC spectra, along with HNCACB, CBCA(CO)NH and HNCO spectra, were recorded at 320.15 K for NHE1-LID and at 278.15 K for NHE1-LID_4G_

The content of secondary structure in LID and 4G was evaluated from C´ and Cα secondary chemical shifts using random coil chemical shifts from POTENCI^35^. The population of transient α-helical structure was estimated from the average SCS value over region divided by 3.09 ppm^83^.

The hydrodynamic radius, *R*_h_, of NHE1-LID was determined from a series of ^1^H,^15^N-HSQC spectra with preceding PGS-LED diffusion filter and with the gradient strength increased linearly from 0.963–47.2 G/cm. To determine the diffusion coefficients, *D*, the decay curves of the amide peaks were plotted against the gradient strength and fitted in Dynamics Center (Bruker) using the equation ($I = I_0 \ast \exp ( - Dx^2\gamma ^2\delta ^2({{\Delta }} - \delta /3)\cdot 10^4)$, with *I* being the intensity of the NMR signal at the respective gradient strength, *I*_0_ the intensity without applied gradient, *x* the gradient strength in G/cm, *γ* = 26752 rad/(G*s), *δ* = 3 ms, Δ = 250 ms. *R*_h_ was calculated from the diffusion coefficient using the Stokes-Einstein relation *R*_h_ = *k*_B_*T*/(6π*ηD*), with *η* being the viscosity of water at the respective temperature.

### Quartz crystal microbalance with dissipation monitoring

QCM-D was performed with a Q-Sense E4 instrument (Q-Sense, Biolin Scientific AB, Sweden), using SiO_2_-coated 5 MHz quartz sensors. Crystals and O-rings were placed in Hellmanex 2% for 10 min, extensively flushed with absolute ethanol and MQ, and then dried under nitrogen flow. Immediately before use, the crystals were treated with a UV ozone cleaner (BioForce Nanosciences, Inc., Ames, IA) for 10 min. Before acquisition, the fundamental frequency and six overtones (3^rd^, 5^th^, 7^th^, 9^th^, 11^th^ and 13^th^) were recorded and the system was equilibrated in MQ at 25 °C, until stable baselines were obtained. After equilibration in buffer, 0.1 mg/mL SUVs POPC:POPS 70 mol%/30 mol% were introduced in the flow cell at 0.2 mL/min and the typical signals for vesicle fusion were followed until a successful bilayer formation was observed.

For homogeneous thin and rigid films fully coupled to the sensor surface, the recorded frequency shifts, normalized to the overtone number, can be simply related to the absorbed mass (*Δm*)^84^. During the experiments, real-time shifts in the resonance frequency (Δ*F*_*n*_) with respect to the calibration value were measured for different overtones indicated as *F*_*n*_, with *n* being the harmonic overtone number. Simultaneously, also the energy dissipation factor (*ΔD*) was evaluated for all the overtones. More details are given in Supplementary Figs. S5–S8.

### Neutron reflectometry (NR)

NR experiments were performed on the SURF reflectometer at ISIS neutron source in Chilton (UK). SURF is a horizontal time of flight reflectometer. Three incoming angles (*θ*) typically of 0.35° and 0.65° and 1.5° were used to cover the *q*-range 8·10^−3^Å^−1^ < *q* < 0.2 Å^−1^, where q is defined as follows:

6 $$q = \frac{{4\pi }}{\lambda }{\mathrm{sin}}(\theta )$$

The measured reflected intensity (*I(q)*) was converted to an absolute reflectivity scale (*R(q)*) by normalization to the direct beam (*I*_*0*_) measured at the same slit settings:

7 $$R\left( q \right) = \frac{{I\left( q \right)}}{{I_0}}$$

The main goal of the NR experiments is to reveal the scattering length density profile (*ρ(z)*) from the experimentally determined reflectivity profiles (Eq. 7). This gives information on the composition of the sample along the surface normal (*z*):

8 $$\rho = \mathop {\sum}\nolimits_i {\frac{{n_ib_i}}{{V_m}}}$$

As reported in Eq. 7, *ρ* depends on the chemical and isotopic composition of the sample as the neutrons are sensitive to the nuclei composing the atoms in the molecules, where *n*_*i*_ is the number of atom *i*, *b*_*i*_ is the coherent scattering length, and *V*_*m*_ is the partial specific molecular volume (also referred as molecular volume). NR was performed using custom-made solid/liquid flow cells with polished silicon crystals (111) with a surface area of 6 ×8 cm. Substrate surfaces were characterized in H_2_O and D_2_O, followed by manual syringe injection of SUVs and incubation for ~30 min to allow for the supported lipid bilayer formation. The membranes were characterized in at least 3 isotopic solvent contrasts, i.e. buffer with different ratio of D_2_O to H_2_O. In particular the buffers used during the experiments were prepared with pure D_2_O (d-buffer, *ρ* = 6.35∙10^−6^ Å^−2^), D_2_O:H_2_O 38:62 w/w (Silicon Matched Water, smw-buffer *ρ* = 2.07∙10^−6^ Å^−2^) and pure H_2_O (h-buffer, *ρ* = −0.56∙10^−6^ Å^−2^). More details are given in Supplementary Figs. S5–S8.

### Construction of an all-atom model of NHE1 LID and simulation systems

An all-atom model of NHE1-LID (C538 – V603) was built using the MOLEFACTURE plugin of VMD^85^. Two helical regions were built in the structure according to information obtained from NMR and secondary structure prediction servers (AGADIR^86^, PSIPRED^87^, JPRED4^88^ as H1 (H545-L562) and H2 (P571- E590). The remaining residues of the model were built as random coil. Hydrogen atoms were automatically added to the protein using the psfgen plugin of VMD. Residues D, E, K and R were charged and histidine residues were neutral. A system was built in which the NHE1-LID model was placed into a 130x130x130 Å^3^ water box with 150 mM NaCl for initial minimization and equilibration (207523 atoms in total) (see below). A structure of the NHE1-LID model (obtained after 500 ps of equilibration) was used to build a second set of systems where NHE1-LID was placed near (~7 Å) the lower leaflet of pre-equilibrated lipid bilayers. The bilayers used were composed of: i) 100% POPC on the upper leaflet (194 molecules) and 70 mol% POPC (150 molecules) and 30 mol% POPS (50 molecules) in the lower leaflet and ii) 100 mol% POPC on the upper leaflet (197 molecules) and 80 mol% POPC (165 molecules) and 20 mol% PI(4,5)P_2_ (33 molecules) in the lower leaflet. Both systems were solvated with randomly placed water molecules and ions (150 mM NaCl) on both sides of the membrane to end up with systems of the following dimensions: 130x130x138 Å^3^ with 163582 atoms and 130x130x142 Å^3^ 169387, respectively.

### Molecular dynamics simulations

MD simulations were performed using GROMACS 2016 and 2018^89^, the CHARMM36m force field for proteins and the TIP3P model for water^90^. The initial system of NHE1-LID on a water box was minimized followed by position restrained simulation in two different phases, NVT and NPT. A 10 ns run of unconstrained NPT equilibration was then performed. We noticed that NHE1-LID promptly (< 1 ns) formed a hairpin structure, so a relaxed but still extended conformation observed after 500 ps was taken for simulations near lipid bilayers (see above). For the system comprising the NHE1-LID model and the POPC/POPC-POPS or POPC/POPC-PI(4,5)P_2_ bilayers a similar protocol was followed. The system was minimized followed by successive rounds of position restrained NVT and NPT relaxations in which constraints to the protein, lipids and solvent atoms were gradually decreased. Finally, NPT unconstrained runs of 860 for the POPC/POPC-POPS system, and 600 ns for the POPC/POPC-PI(4,5)P_2_ system were performed as production runs. The Berendsen thermostat was used for the constrained relaxation runs and the Nose-Hoover thermostat was used for the production runs. In all cases the temperature was 310 K. For the NPT simulations, the Berendsen barostat was used during relaxations and the Parinello-Rahman barostat was used in unconstrained production runs. In all cases the target pressure was 1 atm. A semi-isotropic barostat was used for the simulations with a bilayer. In all the simulations, the verlet-cutoff scheme was used with a 2 fs timestep. A cutoff of 12 Å with a switching function starting at 10 Å was used for non-bonded interactions along with periodic boundary conditions. The Particle Mesh Ewald method was used to compute long-range electrostatic forces. Hydrogen atoms were constrained using the LINCS algorithm^91^. Analysis of the obtained trajectories was performed using VMD plugins, GROMACS analysis tools and in-house prepared tcl and python scripts. All molecular renderings were done with VMD.

### Cell culture and transfection

AP-1 cells^48^ (a Chinese Hamster Ovary (CHO)-derived cell line with no endogenous NHE activity) were grown at 37 °C, 5% CO_2_, 95% humidity in α-minimum Essential medium (Sigma), supplemented with 10% fetal bovine serum, 1% l-glutamine, 1% penicillin/streptomycin and 600 μg/mL Geneticin (G418) sulfate (Invitrogen), and passaged by gentle trypsinization every 3–4 days. Cells were transfected with wt and 4G-variant hNHE1 essentially as described in^92^. Transfectants were selected for resistance to 600 µg/mL G418 (Calbiochem), individual clones picked, and hNHE1 expression verified by immunoblotting and immunofluorescence analysis.

### Biotinylation and immunoblotting

Briefly, cells were grown to ~80% confluence in 10 cm Petri dishes, washed twice in ice-cold PBS, and sulfo-NHS-S-S-biotin (Pierce, #21331) (1.0 mg/mL) was added in ice cold PBS, followed by incubation for 30 min at 4 ^o^C. Cells were washed three times in cold quenching buffer and lysed in cold RIPA buffer (50 mM TrisHCl pH 7.5, 150 mM NaCl, 0.1% SDS, 1 mM sodium orthovanadate, 0.5% sodium deoxycholate, Complete^TM^ mini protease inhibitor tablets (1 tablet/10 mL buffer), 1% Igepal Ca 630, ddH_2_O), incubated for 10 min and scraped off. Lysates were centrifuged for 5 min at 20,000 *g*, 4 ^o^C. The amount of protein in the supernatant was adjusted to 2 µg/µl. 25 µL streptavidine agarose bead solution (Sigma, S1638) were added per 250 µl sample, followed by 2 h of incubation at 4 ^o^C with gentle rolling. Samples were washed 4 times in 1 mL RIPA buffer, centrifuged for 2 min at 2000 *g*, 80 µl IP sample buffer added (100 µl NuPage LDS Sample buffer #NP0007, 0.5 M DTT, ddH_2_O), followed by boiling for 5 min at 95 °C. Samples were vortexed and beads pelleted by centrifugation for 4 min at 15,000 *g*. Identical amounts of sample (20 µg/well) diluted in NuPAGE LDS sample buffer (Lifetech technologies) were boiled for 5 min, separated on NuPage 10% bis-tris gels, and transferred to nitrocellulose membranes using the Novex gel system (Novex, San Diego, CA). Membranes were stained with Ponceau S to confirm equal loading, blocked for 1 h at 37 °C in 120 mM NaCl, 10 mM TrisHCl, 5% nonfat dry milk, and incubated with the primary antibodies against NHE1 (Santa Cruz Biotechnology, sc-136239 (54)) or β-actin (Sigma, A5441) in blocking buffer overnight at 4 °C. After washing in TBS + 0.1% Tween-20 (TBST), membranes were incubated with alkaline phosphatase-conjugated secondary antibodies (1:4000, Sigma), washed, and visualized using BCIP/NBT. Densitometric analysis was performed using UN-SCAN-IT software. Data were plotted using GraphPad Prism 8.4.1 software. For statistical analysis, data from two wt NHE1 clones and two 4G-NHE1 clones, respectively, were pooled to reduce effects of possible clone-to-clone variations.

### Immunofluorescence analysis of NHE1

AP-1 cells (untransfected or stably expressing wt- or 4G-NHE1 as indicated) were grown on 12 mm round glass coverslips, fixed in 2% paraformaldehyde (15 min room temperature, 30 min on ice), washed in TBST, permeabilized for 5 min (0.5% Triton X-100 in TBS), blocked for 30 min (5% BSA in TBST), incubated with primary antibody against NHE1 (Santa Cruz Biotechnology sc-136239 (54); 1:100 dilution in TBST + 1% BSA) o/n at 4 °C. The day after, coverslips were washed 3 times in TBST + 1% BSA and incubated with the appropriate conjugated secondary antibody (1:600 in TBS + 1% BSA) in combination with rhodamine-conjugated phallodin (ThermoFisher, #R415) to label F-actin (1:100 in TBS + 1% BSA) for 1 h, followed by washing in TBST + 1 % BSA, and mounting in N-propyl-gallate mounting medium (2% (w/v) in PBS/glycerol). To stain nuclei, DAPI was added for 3 min following incubation with the secondary antibody. Cells were visualized using the 60X/1.35 NA objective of an Olympus Bx63 epifluorescence microscope. No or negligible labeling was seen in the absence of primary antibody or in untransfected AP-1 cells. Overlays and brightness/contrast adjustment was carried out using Adobe Photoshop software. No other image adjustment was performed.

### Measurements of intracellular pH

Cells were seeded in WillCo dishes (WillCo, Cat. #3522), left to attach o/n, and incubated for 30 min with 2.5 µM 2′,7′-bis-(2-carboxyethyl)-5-(and-6)-carboxyfluorescein (BCECF) acetoxymethyl ester (AM) (BCECF-AM, Invitrogen, #B1150) at 37 °C in a CO_2_ incubator and protected from light. They were washed twice with 37 °C Isotonic Ringer (143 mM NaCl, 5 mM KCl, 1 mM MgSO_4_·7H_2_O, 1 mM Na_2_HPO_4_·2H_2_O, 1 mM CaCl_2_·2H_2_O, 3.3 mM MOPS (3-(N-morpholino)propanesulfonic acid), 3.3 mM TES (2-[Tris(hydroxymethyl)-methylamino]-ethanesulfonic acid), 5 mM HEPES (4-(2-hydroxyethyl)-1-piperazineethanesulfonic acid), adjusted with NaOH to pH 7.4 at 37 °C) and placed in a temperature controlled chamber at 37 °C on a Nikon Eclipse T*i* microscope. Emission intensities after excitation at 440 and 485 nm were simultaneously measured for 10 min in Isotonic Ringer (IR), 10 min in 20 mM NH_4_Cl in IR, 1 min in Na^+^-free Ringer (143 mM NMDG-Cl (N-methyl-D-glucamine chloride), 5 mM KCl, 1 mM MgSO_4_·7H_2_O, 1 mM K_2_HPO_4_·2H_2_O, 1 mM CaCl_2_·2H_2_O, 3.3 mM MOPS, 3.3 mM TES, 5 mM HEPES, adjusted with KOH to pH 7.4 at 37 °C), followed by IR for 10 min to monitor Na^+^-dependent pH_i_ recovery. Calibration using the high K^+^/Nigericin method was performed for each cell line, essentially as in ref. ^93^: cells were consecutively perfused with KCl Ringer (156 mM KCl, 1 mM MgSO_4_·7H_2_O, 1 mM CaCl_2_·2H_2_O, 1 mM K_2_HPO_4_·2H_2_O, 3.3 mM MOPS, 3.3 mM TES, 5 mM HEPES) of pH 6.7, 7.0, 7.2, and 7.4 at 37 °C and 5 µM Nigericin (Sigma, #N-7143) added at each pH. The 485 nm/440 nm BCECF ratio was calculated, and the calibration data was fitted to a linear function in the applied pH range, in which the experimental data (the 485 nm/440 nm ratios) was inserted and converted to corrected pH values. After verifying that the pH_i_ at maximal acidification was not significantly different between cell lines, the recovery rate was determined by fitting a linear line to the initial phase of the pH_i_ recovery. Data were plotted and analyzed using GraphPad Prism 8.4.1 software.

### Statistics and reproducibility

The details about experimental design and statistics used in different data analyses performed in this study are given in the respective sections of results and methods. Pooled cell surface biotinylation data and steady state- and pH_i_, recovery data were normal distributed and of equal variance, and statistical analysis was performed by non-paired, two-sided Student’s *t*-test, with the minimal level of significance *p* < 0.05.

### Reporting summary

Further information on research design is available in the Nature Research Reporting Summary linked to this article.

## Supplementary information

Supplementary Information

Description of Additional Supplementary Files

Supplementary Movie 1

Reporting Summary

## Data Availability

The datasets used and/or analyzed during the current study are available from the corresponding author on reasonable request. Chemical shifts for NHE1-LID have been deposited in the BMRB data base under accession codes 27822 (in MQ) and 27823 (2% LPPG).

## References

[1] Kassem N., et al. Yeast recombinant production of intact human membrane proteins with long intrinsically disordered intracellular regions for structural studies. Biochim. Biophys. Acta (2020). 10.1016/j.bbamem.2020.183272 10.1016/j.bbamem.2020.183272 32169592

[2] Kjaergaard M, Kragelund BB (2017). Functions of intrinsic disorder in transmembrane proteins. Cell. Mol. Life Sci..

[3] Kragelund B. B., Skriver K. Methods in molecular biology: intrinsically disordered proteins. (Springer, 2020). 10.1007/978-1-0716-0524-0_47 35178671

[4] Georgieva ER (2008). Membrane-bound α-synuclein forms an extended helix: long-distance pulsed ESR measurements using vesicles, bicelles, and rodlike micelles. J. Am. Chem. Soc..

[5] Fusco G (2014). Direct observation of the three regions in α-synuclein that determine its membrane-bound behaviour. Nat. Commun..

[6] Cholak E., et al. Distinct α-synuclein:lipid co-structure complexes affect amyloid nucleation through fibril mimetic behaviour. *Biochemistry* (2019). 10.1021/acs.biochem.9b00925 10.1021/acs.biochem.9b00925 31747254

[7] Haxholm GW (2015). Intrinsically disordered cytoplasmic domains of two cytokine receptors mediate conserved interactions with membranes. Biochem. J..

[8] Khan S (2014). Lipotoxic disruption of NHE1 interaction with PI(4,5)P2 expedites proximal tubule apoptosis. J. Clin. Invest.

[9] Phillips R, Ursell T, Wiggins P, Sens P (2009). Emerging roles for lipids in shaping membrane-protein function. Nature.

[10] Bechara C (2015). A subset of annular lipids is linked to the flippase activity of an ABC transporter. Nat. Chem..

[11] Hendus-Altenburger R, Kragelund BB, Pedersen SF (2014). Structural dynamics and regulation of the mammalian SLC9A family of Na+/H+ exchangers. Curr. Top. Membr..

[12] Pedersen SF, Counillon L (2019). The SLC9A-C mammalian Na+/H+ exchanger family: molecules, mechanisms, and physiology. Physiol. Rev..

[13] Orlowski J., Grinstein S. Na ^+^ /H ^+^ Exchangers. In: Comprehensive Physiology. (John Wiley & Sons, Inc., Hoboken, NJ, USA, 2011) 10.1002/cphy.c110020 23733698

[14] Pedersen SF, Stock C (2013). Ion channels and transporters in cancer: pathophysiology, regulation, and clinical potential. Cancer Res..

[15] Wakabayashi S, Fafournoux P, Sardet C, Pouysségur J (1992). The Na+/H+ antiporter cytoplasmic domain mediates growth factor signals and controls "H(+)-sensing&quot. Proc. Natl Acad. Sci. USA.

[16] Ben AmmarY (2006). Crystal structure of CHP2 complexed with NHE1-cytosolic region and an implication for pH regulation. EMBO J..

[17] Mishima M, Wakabayashi S, Kojima C (2007). Solution structure of the cytoplasmic region of Na+/H+ exchanger 1 complexed with essential cofactor calcineurin B homologous protein 1. J. Biol. Chem..

[18] Pang T, Su X, Wakabayashi S, Shigekawa M (2001). Calcineurin homologous protein as an essential cofactor for Na ^+^ /H ^+^ exchangers. J. Biol. Chem..

[19] Li X, Augustine A, Chen S, Fliegel L (2016). Stop codon polymorphisms in the human SLC9A1 gene disrupt or compromise Na+/H+ exchanger function. PLoS ONE.

[20] Ikeda T (1997). Identification of cytoplasmic subdomains that control pH-sensing of the Na+/H+ exchanger (NHE1): pH-maintenance, ATP-sensitive, and flexible loop domains. J. Biochem..

[21] Kjaergaard M., et al. Temperature-dependent structural changes in intrinsically disordered proteins: formation of α-helices or loss of polyproline II? *Protein Sci*. (2010). 10.1002/pro.435 10.1002/pro.435 PMC2923508 20556825

[22] Nørholm A (2011). The intracellular distal tail of the Na+/H+ exchanger NHE1 is intrinsically disordered: implications for NHE1 trafficking. Biochemistry.

[23] Fuchs S (2018). Calcineurin B homologous protein 3 binds with high affinity to the CHP binding domain of the human sodium/proton exchanger NHE1. Sci. Rep..

[24] Grinstein S, Cohen S, Goetz JD, Rothstein A (1985). Osmotic and phorbol ester-induced activation of Na+/H+ exchange: possible role of protein phosphorylation in lymphocyte volume regulation. J. Cell Biol..

[25] Cassel D, Katz M, Rotman M (1986). Depletion of cellular ATP inhibits Na+/H+ antiport in cultured human cells. Modulation of the regulatory effect of intracellular protons on the antiporter activity. J. Biol. Chem..

[26] Abu Jawdeh BG (2011). Phosphoinositide binding differentially regulates NHE1 Na+/H+ exchanger-dependent proximal tubule cell survival. J. Biol. Chem..

[27] Aharonovitz O (2000). Intracellular pH regulation by Na(+)/H(+) exchange requires phosphatidylinositol 4,5-bisphosphate. J. Cell Biol..

[28] Martin TF (1998). Phosphoinositide lipids as signaling molecules: common themes for signal transduction, cytoskeletal regulation, and membrane trafficking. Annu. Rev. Cell Dev. Biol..

[29] Wakabayashi S, Nakamura TY, Kobayashi S, Hisamitsu T (2010). Novel phorbol ester-binding motif mediates hormonal activation of Na+/H+ exchanger. J. Biol. Chem..

[30] Shimada-Shimizu N (2014). Na+/H+ exchanger 1 is regulated via its lipid-interacting domain, which functions as a molecular switch: a pharmacological approach using indolocarbazole compounds. Mol. Pharm..

[31] Shimada-Shimizu N, Hisamitsu T, Nakamura TY, Wakabayashi S (2013). Evidence that Na ^+^ /H ^+^ exchanger 1 is an ATP-binding protein. FEBS J..

[32] Webb BA (2016). A histidine cluster in the cytoplasmic domain of the Na-H exchanger NHE1 confers pH-sensitive phospholipid binding and regulates transporter activity. J. Biol. Chem..

[33] Alexander RT (2011). Membrane surface charge dictates the structure and function of the epithelial Na^+^ /H ^+^ exchanger. EMBO J..

[34] Mohan S (2010). NHE3 activity is dependent on direct phosphoinositide binding at the N terminus of its intracellular cytosolic region. J. Biol. Chem..

[35] Nielsen JT, Mulder FAA (2018). POTENCI: prediction of temperature, neighbor and pH-corrected chemical shifts for intrinsically disordered proteins. J. Biomol. NMR.

[36] Kjaergaard M, Brander S, Poulsen FM (2011). Random coil chemical shift for intrinsically disordered proteins: effects of temperature and pH. J. Biomol. NMR.

[37] Marsh JA, Forman-kay JD (2010). Sequence determinants of compaction in intrinsically disordered proteins. Biophys. J..

[38] Su JY, Hodges RS, Kay CM (1994). Effect of chain length on the formation and stability of synthetic alpha-helical coiled coils. Biochemistry.

[39] Wu L, McElheny D, Huang R, Keiderling TA (2009). Role of tryptophan-tryptophan interactions in Trpzip beta-hairpin formation, structure, and stability. Biochemistry.

[40] Krueger-Koplin RD (2004). An evaluation of detergents for NMR structural studies of membrane proteins. J. Biomol. NMR.

[41] Santiveri CM, Santoro J, Rico M, Jiménez MA (2004). Factors involved in the stability of isolated β-sheets: turn sequence, β-sheet twisting, and hydrophobic surface burial. Protein Sci..

[42] Kyte J, Doolittle RF (1982). A simple method for displaying the hydropathic character of a protein. J. Mol. Biol..

[43] Naumowicz M, Figaszewski ZA (2014). The effect of pH on the electrical capacitance of phosphatidylcholine-phosphatidylserine system in bilayer lipid membrane. J. Membr. Biol..

[44] van Paridon PA, de Kruijff B, Ouwerkerk R, Wirtz KW (1986). Polyphosphoinositides undergo charge neutralization in the physiological pH range: a 31P-NMR study. Biochim. Biophys. Acta.

[45] Höök F. (1997) Development of a Novel QCM Technique for Protein Adsorption Studies. Department of Biochemistry and Biophysics and Department of Applied Physics, Chalmers University

[46] Fragneto-Cusani G (2001). Neutron reflectivity at the solid/liquid interface: examples of applications in biophysics. J. Phys. Condens Matter.

[47] Lind TK, Cárdenas M (2016). Understanding the formation of supported lipid bilayers via vesicle fusion-A case that exemplifies the need for the complementary method approach (Review). Biointerphases.

[48] Rotin D, Grinstein S (1989). Impaired cell volume regulation in Na(+)-H+ exchange-deficient mutants. Am. J. Physiol..

[49] Pedersen SF (2007). NHE1 inhibition by amiloride- and benzoylguanidine-type compounds. Inhibitor binding loci deduced from chimeras of NHE1 homologues with endogenous differences in inhibitor sensitivity. J. Biol. Chem..

[50] Lacroix J, Poët M, Maehrel C, Counillon L (2004). A mechanism for the activation of the Na/H exchanger NHE-1 by cytoplasmic acidification and mitogens. EMBO Rep..

[51] Fafournoux P, Noël J, Pouysségur J (1994). Evidence that Na+/H+ exchanger isoforms NHE1 and NHE3 exist as stable dimers in membranes with a high degree of specificity for homodimers. J. Biol. Chem..

[52] Hisamitsu T, Pang T, Shigekawa M, Wakabayashi S (2004). Dimeric interaction between the cytoplasmic domains of the Na +/H+ exchanger NHE1 revealed by symmetrical intermolecular cross-linking and selective co-immunoprecipitation. Biochemistry.

[53] Boron WF (2004). Regulation of intracellular pH. Adv. Physiol. Educ..

[54] Wang Y, Bugge K, Kragelund BB, Lindorff-Larsen K (2018). Role of protein dynamics in transmembrane receptor signalling. Curr. Opin. Struct. Biol..

[55] Moravcevic K (2010). Kinase associated-1 domains drive MARK/PAR1 kinases to membrane targets by binding acidic phospholipids. Cell.

[56] Lemmon MA (2008). Membrane recognition by phospholipid-binding domains. Nat. Rev. Mol. Cell Biol..

[57] Kutateladze TG (2007). Mechanistic similarities in docking of the FYVE and PX domains to phosphatidylinositol 3-phosphate containing membranes. Prog. Lipid Res..

[58] Rogers JM, Steward A, Clarke J (2013). Folding and binding of an intrinsically disordered protein: Fast, but not “diffusion-limited. J. Am. Chem. Soc..

[59] Dyson HJ, Wright PE (2002). Coupling of folding and binding for unstructured proteins. Curr. Opin. Struct. Biol..

[60] Bugge K (2018). Structure of radical-induced cell death1 Hub domain reveals a common αα-scaffold for disorder in transcriptional networks. Structure.

[61] Reynolds NP (2011). Mechanism of membrane interaction and disruption by α-synuclein. J. Am. Chem. Soc..

[62] Fusco G (2014). Direct observation of the three regions in α-synuclein that determine its membrane-bound behaviour. Nat. Commun..

[63] Xu C (2008). Regulation of T cell receptor activation by dynamic membrane binding of the CD3epsilon cytoplasmic tyrosine-based motif. Cell.

[64] Lecompte M-F, Bouix G, Mannn KG (1994). Electrostatic and Hydrophobic Interactions Are Involved in Factor Va Binding to Membranes Containing Acidic Phospholipids. J Biol Chem.

[65] Aivazian D, Stern LJ (2000). Phosphorylation of T cell receptor zeta is regulated by a lipid dependent folding transition. Nat. Struct. Biol..

[66] Pang V (2012). On the role of the difference in surface tensions involved in the allosteric regulation of NHE-1 induced by low to mild osmotic pressure, membrane tension and lipid asymmetry. Cell Biochem. Biophys..

[67] Lee SA (2005). Targeting of the FYVE domain to endosomal membranes is regulated by a histidine switch. Proc. Natl Acad. Sci. USA.

[68] He J (2008). Molecular mechanism of membrane targeting by the GRP1 PH domain. J. Lipid Res.

[69] Hom RA (2007). pH-dependent binding of the Epsin ENTH domain and the AP180 ANTH domain to PI(4,5)P2-containing bilayers. J. Mol. Biol..

[70] Karlsen ML (2015). Structure of dimeric and tetrameric complexes of the BAR domain protein PICK1 determined by small-angle X-ray scattering. Structure.

[71] Patwardhan P, Resh MD (2010). Myristoylation and membrane binding regulate c-Src stability and kinase activity. Mol. Cell Biol..

[72] Rawat A, Nagaraj R (2010). Determinants of membrane association in the SH4 domain of Fyn: roles of N-terminus myristoylation and side-chain thioacylation. Biochim. Biophys. Acta.

[73] Rawat A, Harishchandran A, Nagaraj R (2013). Fatty acyl chain-dependent but charge-independent association of the SH4 domain of Lck with lipid membranes. J. Biosci..

[74] Hamada K (2000). Structural basis of the membrane-targeting and unmasking mechanisms of the radixin FERM domain. EMBO J..

[75] Bompard G (2003). Membrane targeting of protein tyrosine phosphatase PTPL1 through its FERM domain via binding to phosphatidylinositol 4,5-biphosphate. J. Cell Sci..

[76] Feng J, Mertz B (2015). Novel phosphotidylinositol 4,5-bisphosphate binding sites on focal adhesion kinase. PLoS ONE.

[77] Park M-J (2016). SH2 Domains Serve as Lipid-Binding Modules for pTyr-Signaling Proteins. Mol. Cell.

[78] Sheng R (2016). Lipids regulate Lck protein activity through their interactions with the Lck Src homology 2 domain. J. Biol. Chem..

[79] Muñoz V, Serrano L (1995). Elucidating the folding problem of helical peptides using empirical parameters. III. Temperature and pH dependence. J. Mol. Biol..

[80] Kazimierczuk K, Orekhov VY (2011). Accelerated NMR spectroscopy by using compressed sensing. Angew. Chem. Int. Ed. Engl..

[81] Delaglio F (1995). NMRPipe: A multidimensional spectral processing system based on UNIX pipes. J. Biomol. NMR.

[82] Vranken WF (2005). The CCPN data model for NMR spectroscopy: development of a software pipeline. Proteins.

[83] Spera S, Bax A (1991). Empirical correlation between protein backbone conformation and C.alpha. and C.beta. 13C nuclear magnetic resonance chemical shifts. J. Am. Chem. Soc..

[84] Cans AS (2001). Measurement of the dynamics of exocytosis and vesicle retrieval at cell populations using a quartz crystal microbalance. Anal. Chem..

[85] Humphrey W, Dalke A, Schulten K (1996). VMD: visual molecular dynamics. J. Mol. Graph.

[86] Muñoz V, Serrano L (1997). Development of the multiple sequence approximation within the AGADIR model of α-helix formation: Comparison with Zimm-Bragg and Lifson-Roig formalisms. Biopolymers.

[87] Jones DT (1999). Protein secondary structure prediction based on position-specific scoring matrices. J. Mol. Biol..

[88] Drozdetskiy A, Cole C, Procter J, Barton GJ (2015). JPred4: a protein secondary structure prediction server. Nucleic Acids Res..

[89] Abraham MJ (2015). GROMACS: High performance molecular simulations through multi-level parallelism from laptops to supercomputers. SoftwareX.

[90] Huang J (2017). CHARMM36m: an improved force field for folded and intrinsically disordered proteins. Nat. Methods.

[91] Hess B., Bekker H., Berendsen H. J. C., Fraaije J. G. E. M. LINCS: A Linear Constraint Solver for Molecular Simulations (John Wiley & Sons, Inc, 1997).

[92] Pedersen SF (2007). NHE1 inhibition by amiloride- and benzoylguanidine-type compounds. Inhibitor binding loci deduced from chimeras of NHE1 homologues with endogenous differences in inhibitor sensitivity. J. Biol. Chem..

[93] Pedraz-Cuesta E (2016). Prolactin signaling stimulates invasion via Na(+)/H(+) exchanger NHE1 in T47D human breast cancer cells. Mol. Endocrinol..

[94] Gautier R, Douguet D, Antonny B, Drin G (2008). HELIQUEST: a web server to screen sequences with specific alpha-helical properties. Bioinformatics.

