# Supplementary material for: The intracellular lipid-binding domain of human Na+/H+ exchanger 1 forms a lipid-protein co-structure essential for activity
Source: Commun Biol. 2020 Dec 3;3:731. doi: 10.1038/s42003-020-01455-6 (PMC7713384; doi:10.1038/s42003-020-01455-6)
Supplement: Supplementary file 1 — Supplementary Information [file 42003_2020_1455_MOESM1_ESM.pdf]

**SUPPLEMENTARY**

# The intracellular lipid-binding domain of human $Na^+/H^+$ exchanger 1 forms a lipid-protein co-structure essential for activity

Ruth Hendus-Altenburger[1,2], Jens Vogensen[2], Emilie Skotte Pedersen[1], Alessandra Luchini[3], Raul Araya-Secchi [3], Anne H. Bendsoe[1,2], Nanditha Shyam Prasad[2], Andreas Prestel[1], Marité Cardenas[4], Elena Pedraz Cuesta[2], Lise Arleth[3,#], Stine F. Pedersen[2,#], Birthe B. Kragelund[1,#]

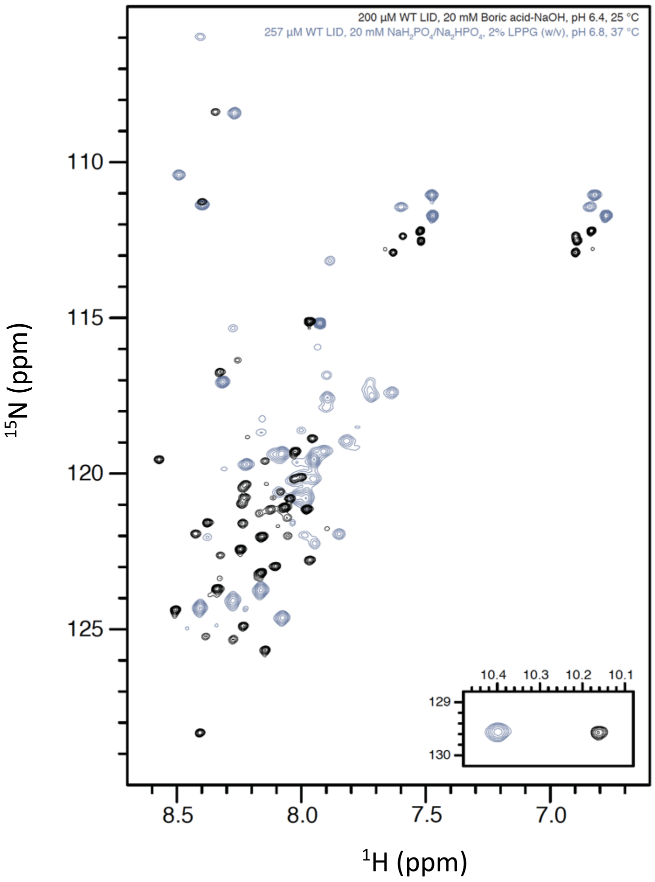


**S1 Fig.** *Comparison of the HSQC spectra of NHE1-LID$_{539\text{-}593}$ in MQ and in LPPG.*

$^{1}$H,$^{15}$N-HSQC spectrum of 200 µM NHE1-LID$_{539\text{-}593}$ in boric acid-NaOH, pH 6.4, 25 °C (black) overlaid the $^{1}$H,$^{15}$N-HSQC spectrum of 257 µM in 20 mM $NaH_2PO_4$,$Na_2HPO_4$, 2% (w/v) LPPG, pH 6.8, 37°. The shift to the right by addition of LPPG is observed in the $^{1}$H-dimension.

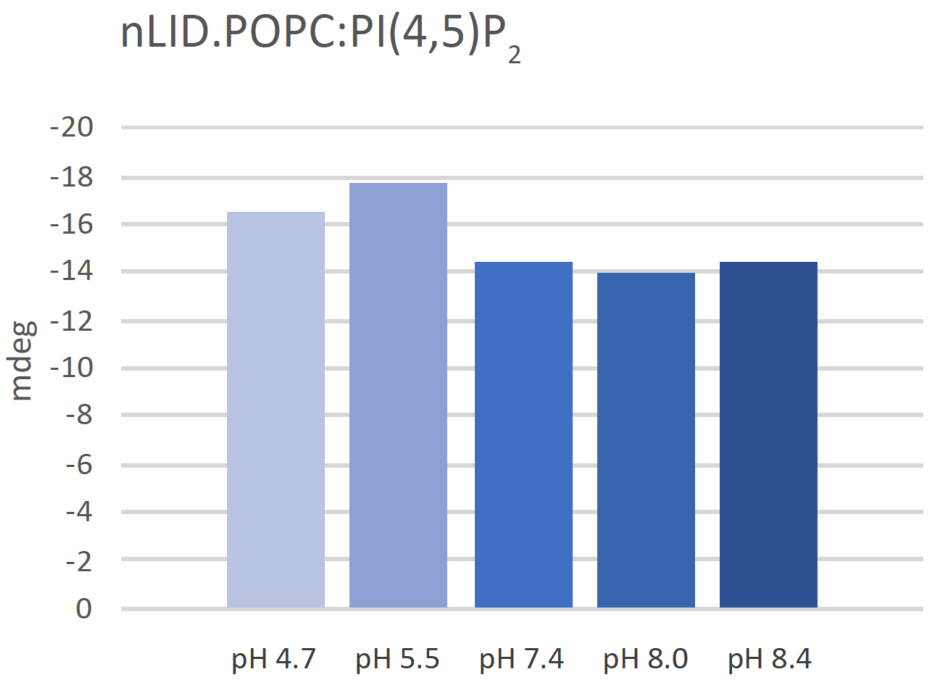


**S2 Fig.** *Changes in helicity of nLID in the presence of POPC:PI(4,5)$P_2$ SUVs as a function of pH measured by far-UV CD.*

The far-UV CD spectra were recorded on a Jasco J-810 spectropolarimeter in a 1 mm quartz cuvette at 298 K on 30 µM nLID with 5mM of POPC:PI(4,5)$P_2$ (95 mol%:5 mol%) SUVs, in 20 mM $NaH_2PO_4$ buffers adjusted with NaOH to the various pH values. Helicity was calculated from the ellipticity at 222 nm as described in methods.

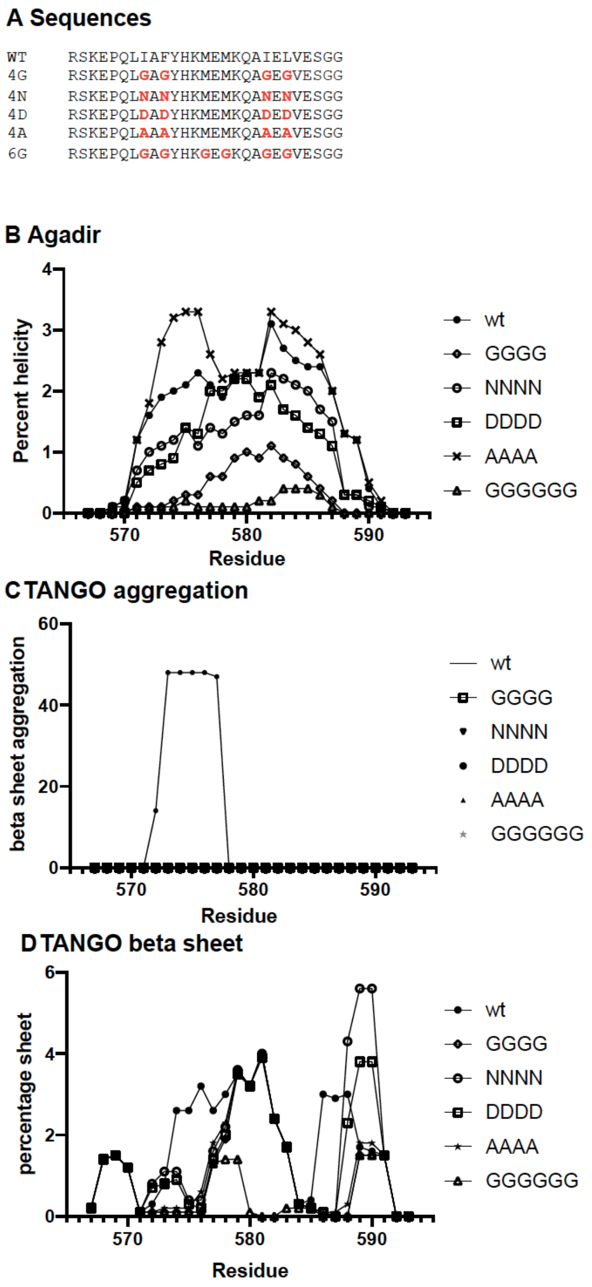


**S3 Fig.** *In silico analysis of the cLID$_{567\text{-}592}$ to suppress aggregation without compromising helicity.*

**(A)** Sequences of the different variants screened. **(B)** AGADIR predictions of helicity. **(C)** TANGO aggregation propensity and **(D)** TANGO beta sheet propensity

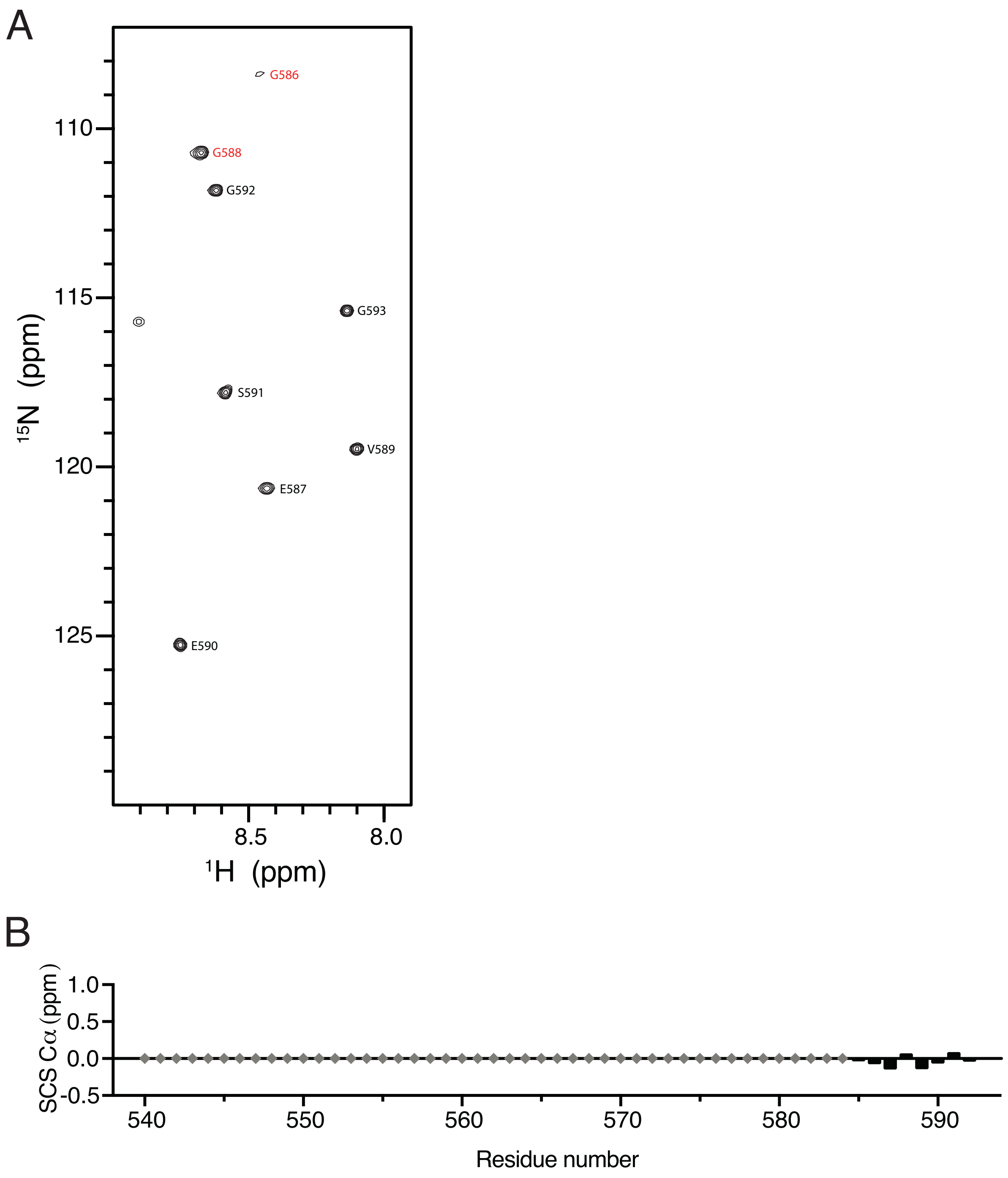


**S4 Fig.** *NMR analysis of NHE1-LID$_{539\text{-}593\text{-}4G}$ in LPPG*

NHE1-LID$_{539\text{-}593\text{-}4G}$ was dissolved in 2% LPPG and recorded at 5 °C to allow for amides in disordered regions to be detected. Backbone assignments were performed from combining HNCACB and CBCA(CO)NH spectra and using previous assignments of NHE1-LID$_{539\text{-}593}$ in water and in the presence of 2% LPPG. **(A)** HSQC spectrum of NHE1-LID$_{539\text{-}593\text{-}4G}$ in 2% LPPG. **(B)** SCS of the assignable residues in NHE1-LID$_{539\text{-}593\text{-}4G}$ in 2% (w/v) LPPG.

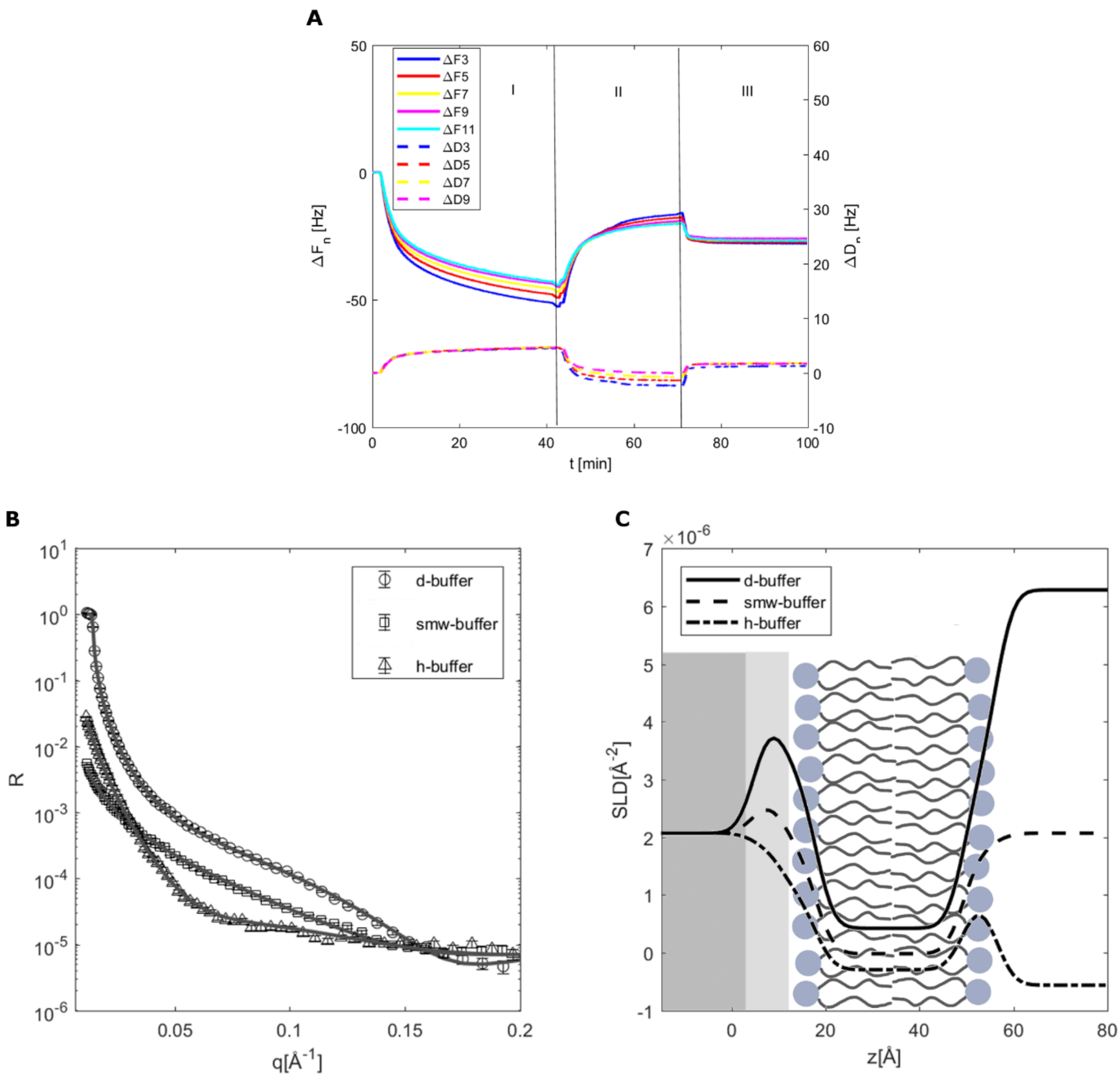


**S5 Fig.** *Supported POPC/POPS bilayer characterization by QCM-D and NR.*

QCM-D measurement on the formation of POPC/POPS (70 mol%:30 mol%) lipid bilayer **(A)**. NR experimental data together with the corresponding fitting curves **(B)**. Data were collected in buffer prepared with different $D_2O$ content (d-buffer=100% $D_2O$, smw-buffer =38%$D_2O$:62% $H_2O$, h-buffer=100% $H_2O$). Scattering length density profile (*ρ(z)*) obtained from NR data analysis **(C)**. Supported lipid bilayer (SLB) formation on the solid substrate was monitored with QCM-D prior to protein solution injection in the QCM-D cell.

Figure S5A-C shows the data collected in the case of POPC/POPS SLB formation as representative of the lipid membrane studied in this work. The time evolution of the frequency shifts (ΔF) and dissipation factors (ΔD) for the different sensor harmonics was divided in three main regions.

After sensor calibration, lipid vesicles in buffer (20mM HEPES, 150mM NaCl, pH=7.4) were injected in the cell (area I). Vesicle adsorption on the sensor surface produced a substantial decrease of the ΔF and increase of ΔD. In order to promote vesicle rupture and formation of a flat lipid bilayer, MQ water was introduced in the cell (area II). The exposure of the adsorbed

vesicles to MQ produced an osmotic shock, which facilitated vesicle rupture and final formation of the supported lipid bilayer (area III). The effective formation of an SLB is characterized by the typical $\Delta F$ value ~ -25Hz. NR provided structural information on the SLBs composed by POPC and POPC/POPS prior to protein solution injection. **S5 B,C Fig** show the NR experimental data together with fitting curves. The collected data were analyzed according to a 4-layer model: 1) silicon oxide layer on the support surface; 2) inner lipid headgroup layer; 3) lipid acyl chains; 4) lipid outer headgroup layer. Preliminary attempts of data fitting highlighted that even in the case of POPC/POPS SLB the two headgroup layers had similar structural parameters. For this reason, during data refinement, the SLBs were considered as symmetric bilayers and the two headgroup layers were linked to have the same structural parameters. The scattering length density profile, $\rho(z)$ together with the optimized structural parameters are reported in **S5C Fig**, and **S1 Table**, respectively.

**S1 Table.** Structural parameters obtained from NR data analysis of plain POPC/POPS bilayers. $t$=thickness, $\rho$= scattering length density; $\phi$=solvent fraction; $\sigma$=roughness.

| Layer | Parameter | POPC/POPS |
|---|---|---|
| *Inner/Outer Headgroup layer* | $t$ [Å] | 7 ± 1 |
| | $\rho_h \cdot 10^{-6}$[Å$^{-2}$] | 1.80 ± 0.01 |
| | $\phi_h$ | 0.30 ± 0.06 |
| | $\sigma_h$[Å] | 3 ± 1 |
| *Acyl tail layer* | $t$ [Å] | 31 ± 1 |
| | $\rho_t$ $10^{-6}$[Å$^{-2}$] | -0.28 ± 0.03 |
| | $\phi_t$ | 0.11 ± 0.01 |
| | $\sigma_t$[Å] | 3 ± 1 |

**S2 Table.** Structural parameters obtained from NR data analysis of the interaction between NHE1-LID and the POPC/POPS lipid membrane.. $t$=thickness, $\rho$= scattering length density; $\phi$=solvent fraction; $\sigma$=roughness. See model fit in figure 5 of main article.

| **Layer** | **Parameter** | **POPC/POPS+NHE1 LID** |
|---|---|---|
| *Inner headgroups* | $t\ [Å]$ | 7 ± 1 |
| | $\rho_h \cdot 10^{-6} [Å^{-2}]$ | 1.80 ± 0.02 |
| | $\phi_h$ | 0.50 ± 0.05 |
| | $\sigma_h [Å]$ | 3 ± 1 |
| *Acyl chains* | $t\ [Å]$ | 30 ± 1 |
| | $\rho_t\ 10^{-6} [Å^{-2}]$ | -0.28 ± 0.03 |
| | $\phi_t$ | 0.09 ± 0.01 |
| | $\sigma_t [Å]$ | 3 ± 1 |
| *Outer headgroups* | $t\ [Å]$ | 11 ± 1 |
| | $\rho_h\ 10^{-6} [Å^{-2}]$ | 1.80 ± 0.01 |
| | $\phi_h$ | 0.19 ± 0.03 |
| | $\sigma_h [Å]$ | 3 ± 1 |
| *Protein layer* | $t\ [Å]$ | 38 ± 3 |
| | $\rho_p\ 10^{-6} [Å^{-2}]$ | 2.6 ± 0.2 (d-buffer)<br>1.9 ± 0.2 (smw-buffer)<br>1.5 ± 0.2 (h-buffer) |
| | $\phi_p$ | 0.81 ± 0.02 |
| | $\sigma_p [Å]$ | 5 ± 1 |

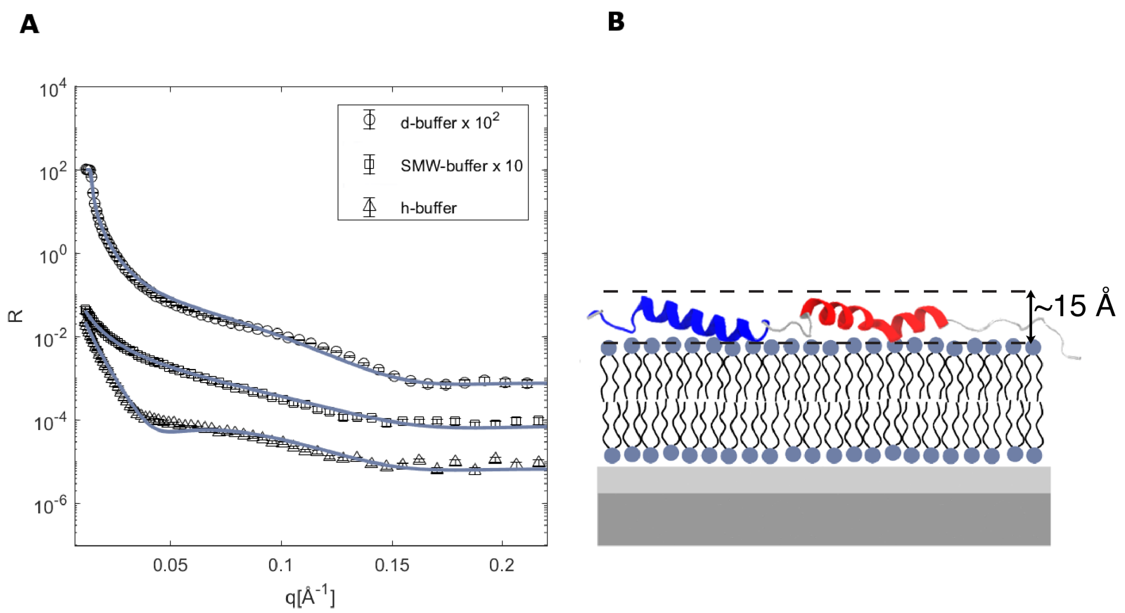


**S6 Fig.** *Simulation of NR-data when assuming an extended conformation of NHE1-LID.*

The NR data collected for NHE1-LID$_{539-593}$ interacting with POPC/POPS bilayer are compared with the theoretical curve calculated for the NHE1-LID$_{539-593}$ being in a fully extended conformation as illustrated in B. In order to establish whether the collected NR data was indeed sensitive to the actual NHE1-LID$_{539-593}$ conformation on the membrane surface, an extended conformation of NHE1-LID$_{539-593}$ was simulated. The protein thickness was calculated as the distance between the helix residues in contact with the lipid headgroup and the ones exposed to the solvent in the opposite direction. This hypothesized structure was used to calculate the theoretical reflectivity curves that are reported in panel A. Clearly the calculated NR curves, with large deviations between model and data, in particular in the h-buffer contrast, are not in good agreement with the experimental data, suggesting that the actual protein layer on the membrane surface is significantly different from the one corresponding to the NHE1-LID$_{539-593}$ in the extended conformation.

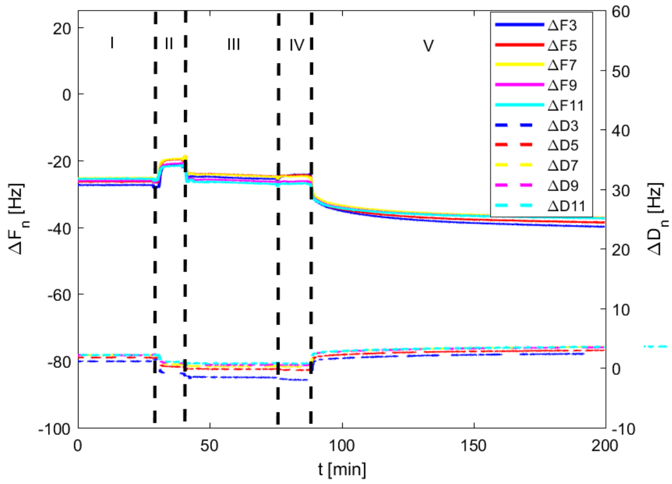


**S7 Fig**. *QCM-D analysis of the interaction with nLID$_{542-569}$ (2.5 µM) and supported POPC/POPS (70 mol%:30 mol%) bilayers.*

Similar to the NHE1 LID (see main text), we also monitored the nLID$_{542-569}$ interaction with lipid membranes with PS lipids (POPC/POPS 70 mol%:30 mol%) with QCM-D. By following the same procedure as described for the NHE1 LID, the formation of the lipid membrane was monitored both in buffer (area I) and in MQ (area II) and subsequently nLID$_{542-569}$ solution (2.5 mM) was injected (area III). The lipid membrane was incubated with the protein solution for about 30 min (area III); the protein excess was removed by flashing MQ (area IV) and the finally the buffer was reintroduced in the cell (area V). The collected data are reported in **S7 Fig** and clearly suggest that even without the C-terminal helix the nLID$_{542-569}$ can still deposit on lipid membranes. Indeed, the frequency shift (ΔF) decreased compared to the signal of the lipid bilayer corresponding to an increase on the adsorbed mass on the sensor surface. Hence, we concluded that nLID$_{542-569}$ molecules were adsorbed on the membrane.

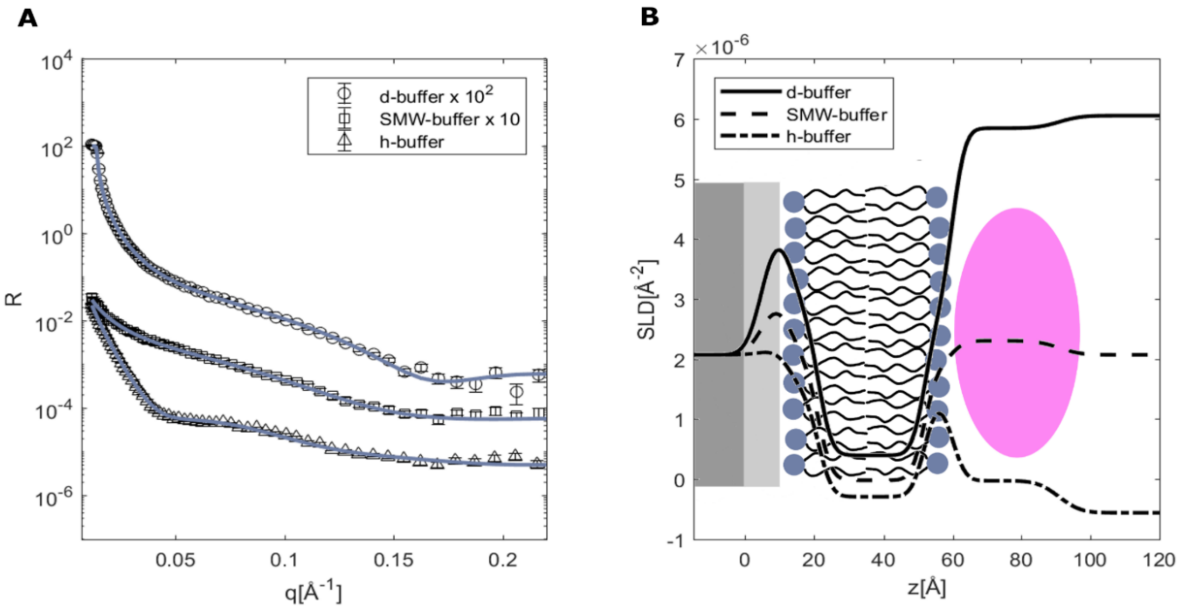


**S8 Fig.** *NHE1-LID$_{539\text{-}593\text{-}4G}$ interaction with lipid membranes (NR)*

NR experimental data together with the corresponding fitting curves for POPC/POPS (70 mol%:30 mol%) lipid bilayer upon injection of NHE1-LID$_{539\text{-}593\text{-}4G}$ **(A)**. Data were collected in buffer prepared with different $D_2O$ content (d-buffer=100% $D_2O$, smw-buffer =38% $D_2O$ 62% $H_2O$, h-buffer=100% $H_2O$). Scattering length density profile (*$\rho(z)$*) obtained from NR data analysis **(B)**. We measured the interaction of the NHE1-LID$_{539\text{-}593\text{-}4G}$ with lipid membranes (POPC/POPS 70 mol%:30 mol%) by NR. The formation of the supported lipid membrane was initially characterized and subsequently the NHE1-LID$_{539\text{-}593\text{-}4G}$ solution (2.5μM) was injected. The same protocol described in the main text for the NHE1-LID$_{539\text{-}593}$ was used in this case as well. NR data were successfully analyzed by applying the same model described for the NHE1-LID$_{539\text{-}593}$ (**Fig. 5. Main text**). Hence, also in this case protein molecules interacting with the membrane surface were observed. Interestingly, in the case of the NHE1-LID$_{539\text{-}593\text{-}4G}$ the membrane bilayer structure was found to be unperturbed. Indeed, while in the case of the NHE1-LID$_{539\text{-}593}$ the structural parameters of the membrane headgroup layer where found considerably affected by the protein-membrane interaction, in the case of NHE1-LID$_{539\text{-}593\text{-}4G}$ such an effect was not observed. This result suggests that the NHE1-LID$_{539\text{-}593\text{-}4G}$ is not as closely associated to the membrane as the NHE1-LID$_{539\text{-}593}$. The structural parameters obtained from the NHE1-LID$_{539\text{-}593\text{-}4G}$ data analysis are summarized in **S3 Table**. As in the case of NHE1-LID, data analysis was based on a 5 layers model, where a layer was used for describing the silicon oxide present on the substrate surface, 3 layers were used to describe the lipid bilayer structure (two outer lipid headgroup layers and one intermediate acyl chain layer). Finally, the NHE1-LID$_{539\text{-}593\text{-}4G}$ molecules interacting with the membrane were described as a highly hydrated protein layer adsorbed on the membrane surface. Compared to NHE1-LID$_{539\text{-}593}$, the protein layer thickness in the case of NHE1-LID$_{539\text{-}593\text{-}4G}$ resulted to be considerably reduced. This result suggests that the mutations introduced in the NHE1-LID$_{539\text{-}593}$ structure are responsible for a remarkable conformation change in the protein structure next to the lipid membrane.

**S3 Table.** Structural parameters obtained from NR data analysis of the NHE1-$LID_{539-593-4G}$. $t$=thickness, $\rho$= scattering length density; $\phi$=solvent fraction; $\sigma$=roughness.

| Layer | Parameter | POPC/POPS+NHE1 $LID_{4G}$ |
|---|---|---|
| *Inner headgroups* | $t$ [Å] | 9 ± 1 |
| | $\rho_h \cdot 10^{-6}$[Å$^{-2}$] | 1.80 ± 0.01 |
| | $\phi_h$ | 0.20 ± 0.05 |
| | $\sigma_h$[Å] | 3 ± 1 |
| *Acyl chains* | $t$ [Å] | 28 ± 1 |
| | $\rho_t$ $10^{-6}$[Å$^{-2}$] | -0.28 ± 0.03 |
| | $\phi_t$ | 0.07 ± 0.01 |
| | $\sigma_t$[Å] | 3 ± 1 |
| *Outer headgroups* | $t$ [Å] | 9 ± 1 |
| | $\rho_h$ $10^{-6}$[Å$^{-2}$] | 1.80 ± 0.01 |
| | $\phi_h$ | 0.20 ± 0.05 |
| | $\sigma_h$[Å] | 3 ± 1 |
| *Protein layer* | $t$ [Å] | 28 ± 4 |
| | $\rho_p$ $10^{-6}$[Å$^{-2}$] | 2.6 ± 0.2 (d-buffer)<br>1.9 ± 0.2 (smw-buffer)<br>1.5 ± 0.2 (h-buffer) |
| | $\phi_p$ | 0.83 ± 0.04 |
| | $\sigma_p$[Å] | 5 ± 1 |

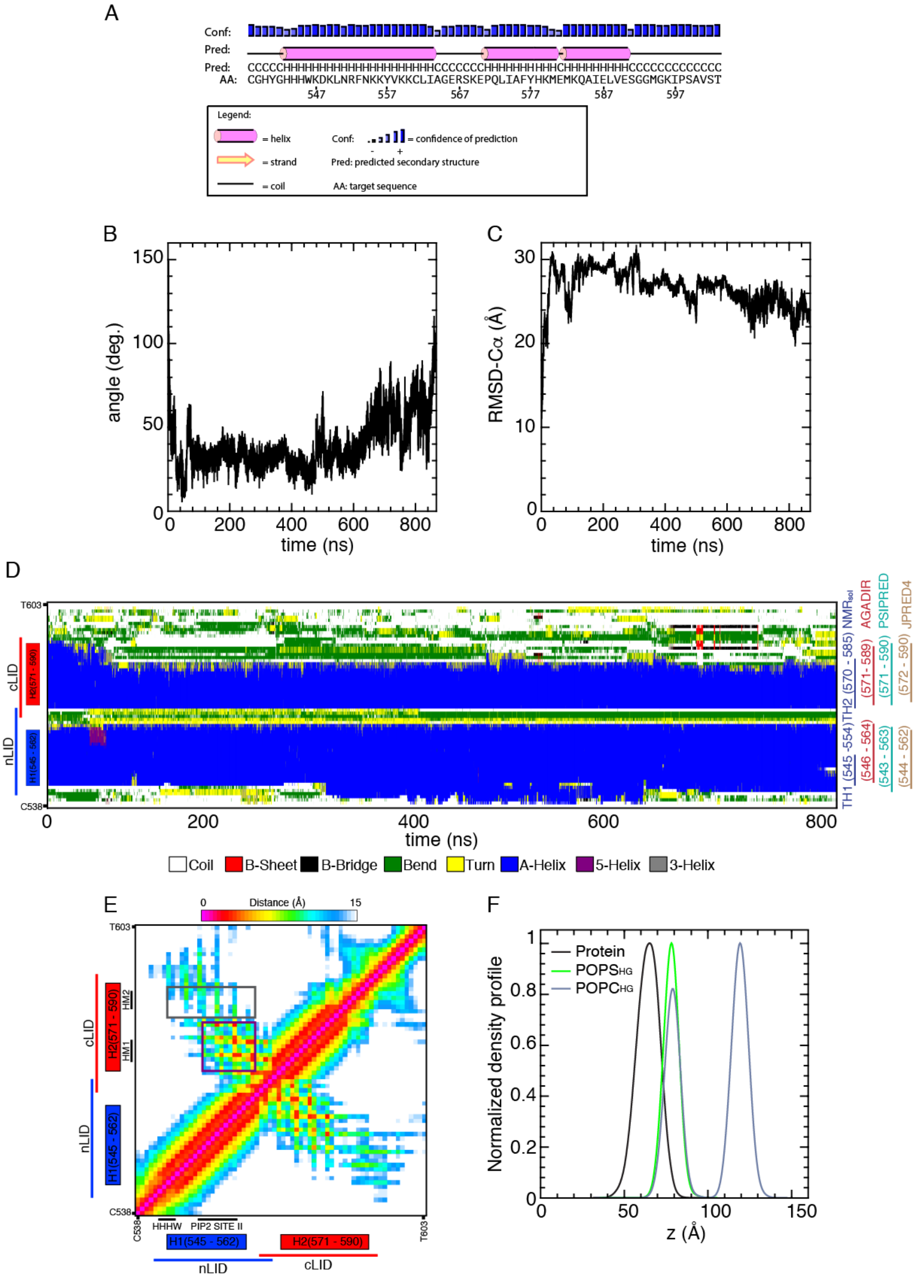


**S9 Fig.** *Molecular dynamics simulation of NHE1 LID near a POPC/POPS bilayer.*

**(A)** Secondary structure prediction for NHE1 LID (538 – 603) using PSIPRED [1]. **(B)** Angle between the principal axes of the helices H1 and H2 of NHE1 LID during the simulation. **(C)** RMSD-Cα of NHE1 LID during the simulation. The initial NHE1 LID model (see methods) was used as reference. **(D)** Temporal evolution of secondary structure elements in NHE1 LID observed during the simulation. On the right different definitions of the helical regions attributed to NHE1 LID obtained from NMR and from different prediction methods (AGADIR [2], PSIPRED [1], JPRED4 [3]. **(E)** Average contact map obtained from the simulation. The purple box highlights regions of inter-helical contacts, involving the C-terminal half of H1 and N-terminal half of H2. The gray box highlights the C-terminal half of H2 where less protein-protein contacts are formed. Remarkably, the residues that form the PI(4,5)$P_2$ binding site (PI(4,5)$P_2$ SITE - II) on H1 form the main contacts with the N-terminal half of H2, including

some residues from HM1. On the other hand, hydrophobic residues on the C-terminal half of H2 (HM2 and neighboring residues) do not engage in protein-protein contacts participating instead in contacts with the membrane (**Fig 5C-D**). **(F)** Normalized average density profiles for the protein and the headgroups of POPC and POPS obtained from the simulation (omitting the first 100 ns of the trajectory).

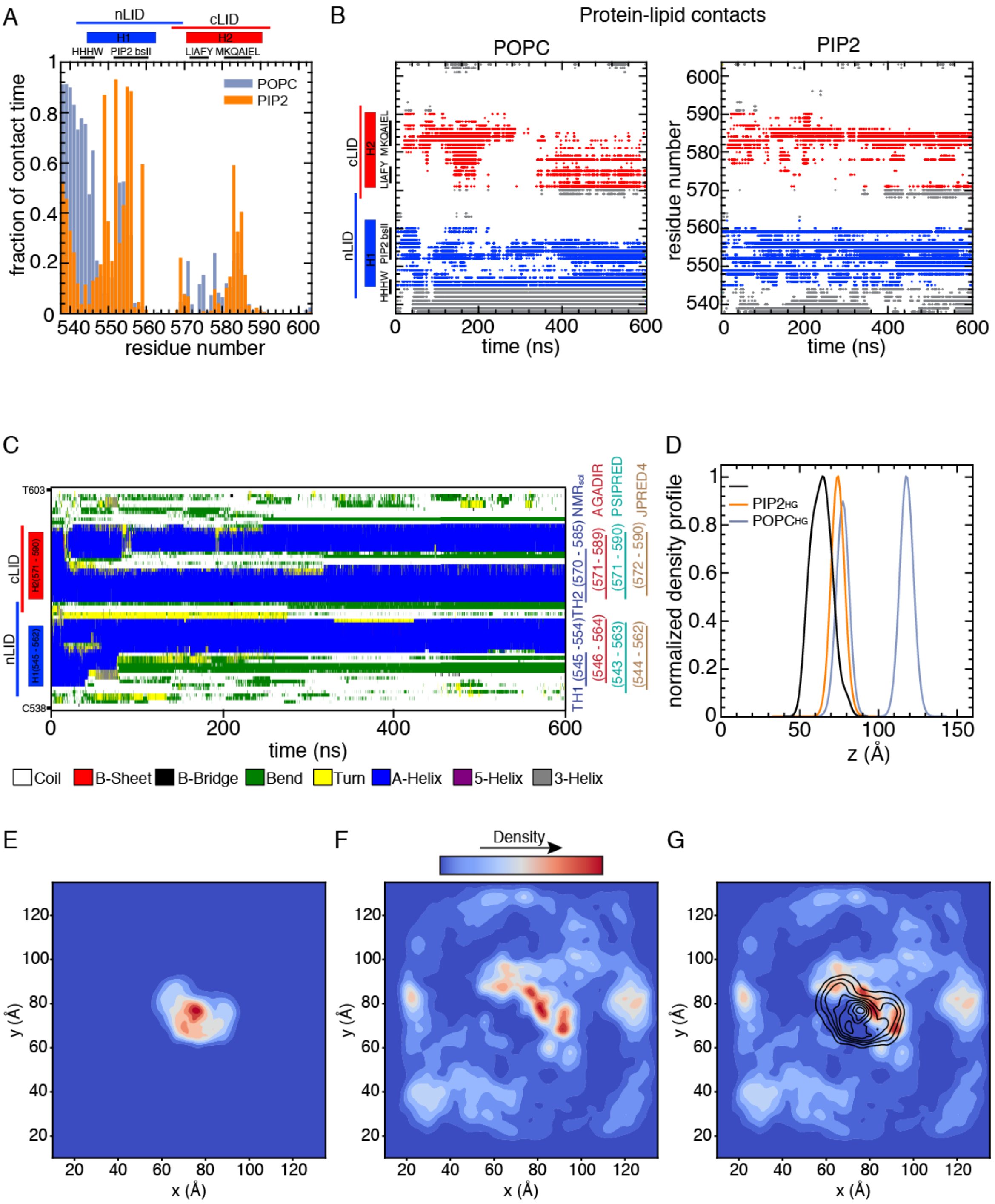


**S10 Fig.** *Molecular dynamics simulation of NHE1 LID near a POPC/PI(4,5)$P_2$ bilayer*

**(A)** Per-residue histogram of protein-lipid contacts observed during the MD simulation (blue-grey: POPC, orange: PI(4,5)$P_2$). **(B)** Temporal evolution of protein-POPC and protein-PI(4,5)$P_2$ contacts from the MD simulation. **(C)** Temporal evolution of secondary structure elements in NHE1 LID observed during the simulation. On the right different definitions of the helical regions attributed to NHE1 LID obtained from NMR (and from different prediction

methods (AGADIR [2], PSIPRED [1], JPRED4 [3]). (**D**) Normalized average density profiles for the protein and the headgroups of POPC and PI(4,5)$P_2$ obtained from the simulation (omitting the first 100 ns od the trajectory). **(E-F)** Normalized average density maps for the protein (E) and the headgroups of PIP2 (F) obtained from the simulation (omitting the first 100 ns of the trajectory). (**G**) Superimposition of the contours from E on F to highlight the accumulation of PI(4,5)$P_2$ lipids around the NHE1-LID protein.

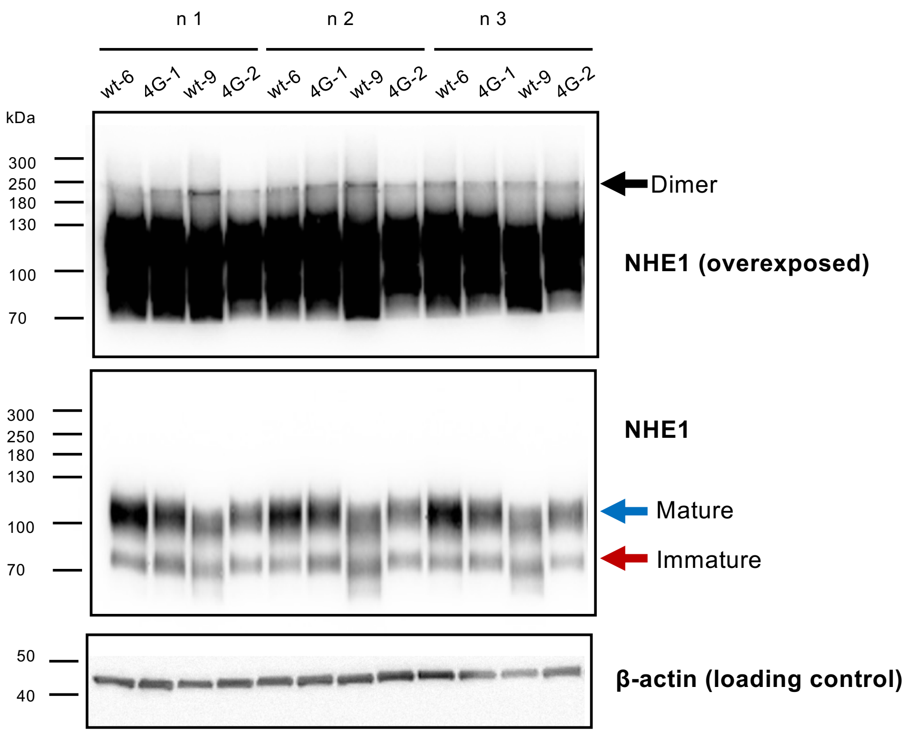


**S11 Fig.** *NHE1 dimer formation is not detectably affected by 4G mutation.*

The figure shows 3 representative immunoblots of lysates of AP-1 cells expressing each of the two wt and 4G variants, respectively. The top band is overexposed to reveal the dimer band, indicated by a black arrow. Fully (mature, blue arrow) and partially (immature, red arrow) NHE1 is indicated on the middle blot. β-actin is shown as loading control.

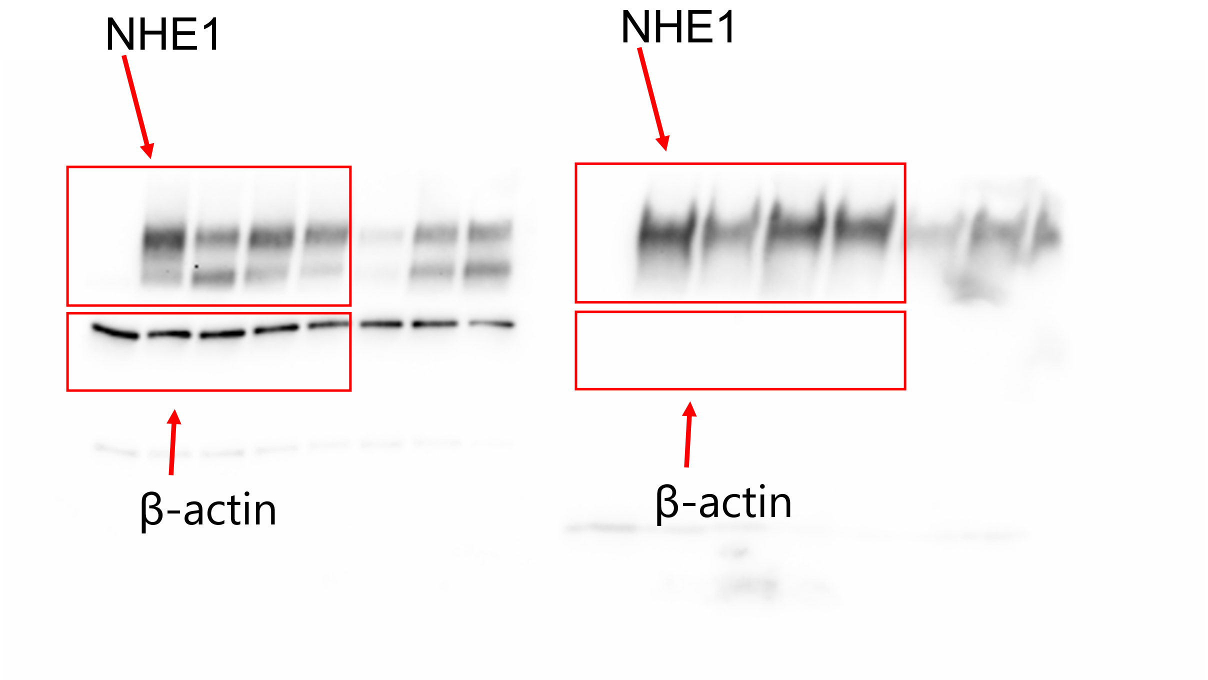


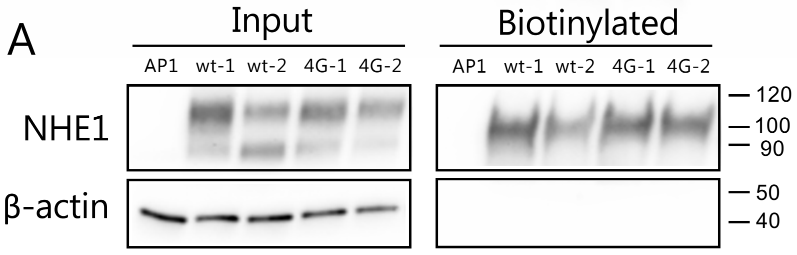


**S12.** ***Uncropped blots for Fig 6a.***

The figure shows the uncropped, unadjusted blots from Fig. 6a. Red boxes mark the relevant lanes and blots. The final figure is shown below for comparison
