# Supplementary material for: The intracellular lipid-binding domain of human Na+/H+ exchanger 1 forms a lipid-protein co-structure essential for activity
Source: Commun Biol. 2020 Dec 3;3:731. doi: 10.1038/s42003-020-01455-6 (PMC7713384; doi:10.1038/s42003-020-01455-6)
Supplement: Supplementary file 2 — Description of Additional Supplementary Files [file 42003_2020_1455_MOESM2_ESM.pdf]

Description of Additional Supplementary Files

Title: Supplementary movie 1:

Description: Movie taken from the MD simulation of NHE1-LID on a POPC:POPS bilayer depicting the time evolution of bound orientations of NHE1-LID on the membrane. NHE1-LID is shown in ribbon representation, lipids are shown in van der Waals' representation (hydrogen atoms omitted for clarity), Helix H1, blue; helix H2, red; POPC blue-grey; POPS, green)
