# Supplementary material for: The intracellular lipid-binding domain of human Na+/H+ exchanger 1 forms a lipid-protein co-structure essential for activity
Source: Commun Biol. 2020 Dec 3;3:731. doi: 10.1038/s42003-020-01455-6 (PMC7713384; doi:10.1038/s42003-020-01455-6)
Supplement: Supplementary file 4 — Reporting Summary [file 42003_2020_1455_MOESM4_ESM.pdf]

nature research

Corresponding author(s): Birthe B. Kragelund

Last updated by author(s): Apr 9, 2020

Reporting Summary

Nature Research wishes to improve the reproducibility of the work that we publish. This form provides structure for consistency and transparency in reporting. For further information on Nature Research policies, see Authors & Referees and the Editorial Policy Checklist .

Statistics

For all statistical analyses, confirm that the following items are present in the figure legend, table legend, main text, or Methods section.

n/a	Confirmed	
[ ]	[x]	The exact sample size (*n*) for each experimental group/condition, given as a discrete number and unit of measurement
[ ]	[x]	A statement on whether measurements were taken from distinct samples or whether the same sample was measured repeatedly
[ ]	[x]	The statistical test(s) used AND whether they are one- or two-sided<br>*Only common tests should be described solely by name; describe more complex techniques in the Methods section.*
[x]	[ ]	A description of all covariates tested
[ ]	[x]	A description of any assumptions or corrections, such as tests of normality and adjustment for multiple comparisons
[ ]	[x]	A full description of the statistical parameters including central tendency (e.g. means) or other basic estimates (e.g. regression coefficient) AND variation (e.g. standard deviation) or associated estimates of uncertainty (e.g. confidence intervals)
[ ]	[x]	For null hypothesis testing, the test statistic (e.g. *F*, *t*, *r*) with confidence intervals, effect sizes, degrees of freedom and *P* value noted<br>*Give P values as exact values whenever suitable.*
[x]	[ ]	For Bayesian analysis, information on the choice of priors and Markov chain Monte Carlo settings
[x]	[ ]	For hierarchical and complex designs, identification of the appropriate level for tests and full reporting of outcomes
[x]	[ ]	Estimates of effect sizes (e.g. Cohen's *d*, Pearson's *r*), indicating how they were calculated

Our web collection on statistics for biologists contains articles on many of the points above.

Software and code

Policy information about availability of computer code

Data collection	PTI international proprietary data aquisition software; Olympus Cellsens software; RasCAL; Topspin;
Data analysis	GraphPad Prism 8.4.1; Adobe photoshop; Excel; UN-SCAN-IT densitometry software; VMD; Gromacs v2016 and v2018; CcpNMR Analysis; qMMD; NMRDraw; NMRPipe; Potensti; Jasco CD analysis software;

For manuscripts utilizing custom algorithms or software that are central to the research but not yet described in published literature, software must be made available to editors/reviewers. We strongly encourage code deposition in a community repository (e.g. GitHub). See the Nature Research guidelines for submitting code & software for further information.

Data

Policy information about availability of data

All manuscripts must include a data availability statement. This statement should provide the following information, where applicable:
- Accession codes, unique identifiers, or web links for publicly available datasets
- A list of figures that have associated raw data
- A description of any restrictions on data availability

Fig. 1,2,3,5 and 6 have asssociated raw data;

Field-specific reporting

Please select the one below that is the best fit for your research. If you are not sure, read the appropriate sections before making your selection.

[x] Life sciences [ ] Behavioural & social sciences [ ] Ecological, evolutionary & environmental sciences

October 2018

Life sciences study design

All studies must disclose on these points even when the disclosure is negative.

Sample size	Sample size was based on an estimate of the effect size, based on previous comparable experiments.
Data exclusions	No data were excluded from analysis.
Replication	All experiments on cells were independently repeated at least 3 times.
Randomization	N/R
Blinding	Immunofluoresence data were blinded when acquiring microscopy images. All other data are quantitative and blinding not relevant.

Reporting for specific materials, systems and methods

We require information from authors about some types of materials, experimental systems and methods used in many studies. Here, indicate whether each material, system or method listed is relevant to your study. If you are not sure if a list item applies to your research, read the appropriate section before selecting a response.

Materials & experimental systems

n/a	Involved in the study	
☐	☒	Antibodies
☐	☒	Eukaryotic cell lines
☒	☐	Palaeontology
☒	☐	Animals and other organisms
☒	☐	Human research participants
☒	☐	Clinical data

Methods

n/a	Involved in the study	
☒	☐	ChIP-seq
☒	☐	Flow cytometry
☒	☐	MRI-based neuroimaging

Antibodies

Antibodies used	anti-NHE1: Santa Cruz Biotechnology, sc-136239, clone 54; anti-beta-actin: Sigma, A5441
Validation	The anti-NHE1 antibody has been extensively validated by use of NHE1 knockout-, knockdown- and overexpressing cell lines in multiple papers published by our laboratory, and as seen in Fig. 6A, AP-1 cells which lack NHE1 are devoid of staining with this antibody. Fig. 6A also shows that biotinylated fractions, which lack beta-actin, are devoid of staining with the beta-actin antibody used as loading control.

Eukaryotic cell lines

Policy information about cell lines

Cell line source(s)	AP-1 cells, a CHO-derived cell line lacking endogenous NHE activity. Cells are derived in the laboratory of Sergio Grinstein, University of Toronto, and a kind gift from this laboratory. AP-1 cells stably expressing wild type and mutant NHE1 are generated in our laboratory.
Authentication	All experiments were accompanied by parallel western blots to determine NHE1 expression
Mycoplasma contamination	All cells used were tested for mycoplasma infection every 3 months and found negative
Commonly misidentified lines (See ICLAC register)	N/A
